# Combined metagenomic and metabolomic analyses reveal that *Bt* rice planting alters soil C-N metabolism

**DOI:** 10.1038/s43705-023-00217-9

**Published:** 2023-01-23

**Authors:** Peng Li, Shuifeng Ye, Jun Chen, Luyao Wang, Yujie Li, Lei Ge, Guogan Wu, Lili Song, Cui Wang, Yu Sun, Jinbin Wang, Aihu Pan, Zhexue Quan, Yunfei Wu

**Affiliations:** 1 Shanghai Key Laboratory of Agricultural Genetics and Breeding, Biotechnology Research Institute, Shanghai Academy of Agricultural Sciences, 201106 Shanghai, China; 2 Shanghai Co-Elite Agricultural Sci-Tech (Group) Co., Ltd, 201106 Shanghai, China; 3 College of Life Sciences, Shangrao Normal University, 334001 Shangrao, China; 4 East China University of Technology, 330013 Nanchang, China; 5 School of Life Sciences, Fudan University, 200433 Shanghai, China; 6 The College of Bioscience and Biotechnology, Yangzhou University, 225009 Yangzhou, China

**Keywords:** Microbial ecology, Microbiome

## Abstract

The environmental impacts of genetically modified (GM) plants remain a controversial global issue. To address these issues, comprehensive environmental risk assessments of GM plants is critical for the sustainable development and application of transgenic technology. In this paper, significant differences were not observed between microbial metagenomic and metabolomic profiles in surface waters of the *Bt* rice (T1C-1, the transgenic line) and non-*Bt* cultivars (Minghui 63 (the isogenic line) and Zhonghua 11 (the conventional *japonica* cultivar)). In contrast, differences in these profiles were apparent in the rhizospheres. T1C-1 planting increased soil microbiome diversity and network stability, but did not significantly alter the abundances of potential probiotic or phytopathogenic microorganisms compared with Minghui 63 and Zhonghua 11, which revealed no adverse effects of T1C-1 on soil microbial communities. T1C-1 planting could significantly alter soil C and N, probably via the regulation of the abundances of enzymes related to soil C and N cycling. In addition, integrated multi-omic analysis of root exudate metabolomes and soil microbiomes showed that the abundances of various metabolites released as root exudates were significantly correlated with subsets of microbial populations including the Acidobacteria, Actinobacteria, Chloroflexi, and Gemmatimonadetes that were differentially abundant in T1C-1 and Mnghui 63 soils. Finally, the potential for T1C-1-associated root metabolites to exert growth effects on T1C-1-associated species was experimentally validated by analysis of bacterial cultures, revealing that *Bt* rice planting could selectively modulate specific root microbiota. Overall, this study indicate that *Bt* rice can directly modulate rhizosphere microbiome assemblages by altering the metabolic compositions of root exudates that then alters soil metabolite profiles and physiochemical properties. This study unveils the mechanistic associations of *Bt* plant-microorganism-environment, which provides comprehensive insights into the potential ecological impacts of GM plants.

## Introduction

The first wide-scale commercial planting of genetically modified (GM) crops occurred with *Bt* maize in 1996 [1]. The estimated global commercial growing area for GM crops in 2019 comprised 190.4 million hectares and crops with insect resistance via *Bacillus thuringiensis* (*Bt*) toxin are now dominant among these [2]. Rice is one of the most important crops globally and is a primary food source for over half of the global population [3]. *Bt* rice express insecticidal toxin from the bacterium *Bacillus thuringiensis* (*Bt*), allowing strong resistance to stem-borers and leaf-folders, which are two major pests of rice [4]. The secretion through root exudates of *Bt* crops is the main way for Bt toxin to enter the soil ecosystem [5]. Bt toxins that are produced from genes engineered by artificial modification may thus potentially enter the rhizosphere as an exogenous toxic compound that interacts with native soil microorganisms [6, 7]. The rapid increase in planting of *Bt* crops has led to global environmental concerns for applications related to *Bt* crops [8]. Therefore, environmental risk assessments must be implemented and identified prior to planting GM crops like *Bt* rice in order to assess whether detrimental effects on agroecosystems are likely to occur [9, 10].

One of the major potential environmental risks associated with the use of *Bt* crops are the effects on soils and soil-inhabiting non-target organisms, including beneficial soil bacteria and fungi [11]. Bacterial and fungal communities are components of rhizosphere ecosystems that include the soils influenced by root metabolites. These ecosystems are critical areas for biogeochemical transformations like nitrogen fixation and nutrient cycling [12–14]. Further, microbial composition can shape environmental biochemistry in the rhizosphere through microbial metabolites involved in nitrogen, amino acid, and carbohydrate metabolism [14, 15]. Accordingly, soil metabolites can be regarded as phenotypes or signatures of soil microbial community changes because alterations at the organismal and enzymatic levels will manifest as altered metabolite profiles [16]. Broadly, rhizosphere metabolite profiles are derived from root exudates, indigenous soil metabolites, and microbially-derived compounds that together shape microbiota-plant interactions [17]. However, rhizosphere communities are also likely influenced by potentially harmful root exudates, including Bt toxins released from *Bt* crops. Accordingly, rhizosphere metabolomics can potentially help better understand chemical communications between root and rhizosphere microorganisms.

Our previous study revealed that the total level of organic acids in root exudates were significantly different between *Bt* cotton and its conventional parental variety [8]. Thus, it is likely that wide variation in the quantity and quality of root exudates can be attributed to the introduction of *Bt* genes and these dynamics might affect rhizosphere metabolite profiles and rhizosphere microbial community assembly. However, it is less clear how specific microorganisms and the metabolites they produce could change based on changes in root exudates from *Bt* plants compared with conventional plant varieties. In addition, water environments may also be particularly affected by exudates from aquatic plants since these are key places where plants and microorganisms co-exist and engage in symbiotic relationships critical to ecosystem functions. For example, Rosi-Marshall [18] and Tank [19] reported that *Bt* maize detritus and associated Bt toxins were widely distributed and persistent in headwater streams, suggesting that *Bt* maize byproducts may affect headwater stream ecosystems. However, the effects of *Bt* crops on waterborne microbial communities and metabolites have not yet been examined, and this is especially true for semi-aquatic plants like *Bt* rice. Such investigations may provide additional insights into the potential effects of *Bt* introduction on non-target organisms.

Several studies have suggested that changes in microbial community structures of *Bt* plants exhibited negligible differences compared to communities of conventional varieties [6, 20]. Nevertheless, these results have been challenged by subsequent studies with contrasting results [21, 22]. To understand the comprehensive impacts of *Bt* rice cultivation on agroecosystems, accurate information on microbial community structures, functional potentials, and metabolic characteristics are needed. Deep shotgun sequencing and metagenome-wide association studies have accordingly enabled more in-depth characterization and insights into the taxonomic and functional diversity of soil microbiomes compared to traditional methods including cultivation-based, fluorescence in situ hybridization, microarray, or 16 S rRNA gene amplicon sequencing [23–25]. Metabolomics provides detailed metabolite profiling information and allows the identification of differentially enriched metabolic pathways that can help reveal mechanisms underpinning the interplay between microbial communities and environments [24]. Moreover, soil metabolomes can contain information for hundreds of analytes including amino acids, carbohydrates, lipids, and organoheterocyclic compounds, thereby confirming changes to soil molecular pools including carbon and nitrogen pools [14, 26]. Thus, changes in microbiomes and metabolomes at the plant-microbe-environment interface can reflect integrated responses of both microorganisms and the biochemistry of their ecosystems in association with GM plants. Nevertheless, these dynamics are not well understood. In particular, combined bio-omic (e.g., metagenomics and metabolomics) investigative approaches have not been applied to understand the putative mechanistic associations between *Bt* rice-linked soil or water microorganisms and metabolites.

**Fig. 1:**
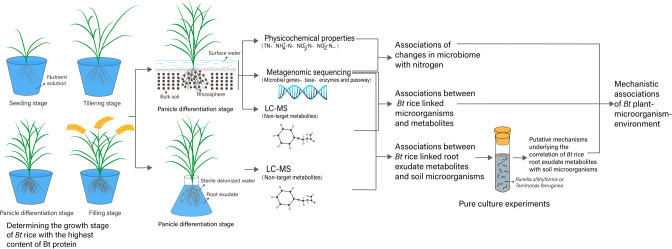
Flow chart of experimental procedures.

Root exudates vary with plant development [27] and thus, an investigation performed at specific development stages associated with the highest contents of Bt toxin in root exudates may provide particularly valuable information for environmental risk assessments of *Bt* plants. Such studies would be especially useful for connecting information of soil microbiome characteristics with soil metabolite and root exudate profiles. In this study, *Bt* rice (T1C-1), its parental non-transgenic cultivar (Minghui 63), and a conventional cultivar (Zhonghua 11) were cultivated in nutrient solutions to determine the plant growth stage at which Bt toxin concentrations from *Bt* rice were highest. Shotgun metagenomic sequencing was also performed in addition to untargeted liquid chromatography-mass spectrometry (LC-MS) metabolomic profiling of soil and surface water samples to identify microorganisms, enzymes, and metabolites that were differentially abundant in *Bt* rice soils compared to parental cultivars. Significantly different biological pathways between *Bt* rice and its parental rice cultivar were identified based on KEGG pathway enrichment analysis. We specifically focused on investigating putative mechanistic associations between *Bt* rice-associated soil microorganisms and metabolites. Finally, correlations between root exudates and rhizosphere microorganisms were evaluated, in addition to growth responses of *Bt* rice-associated microorganisms in rhizosphere to *Bt* rice-associated metabolites in root exudates. These results were used to investigate whether direct manipulation of soil microbiomes by *Bt* rice was accomplished due to the specific composition of root exudates that leads to further alteration of soil metabolite profiles (Fig. 1).

## Materials and methods

### Plants and experimental design

The T1C-1 rice cultivar is bred by introducing the *Cry1C* gene encoding *Bacillus thuringiensis* (*Bt*) delta endotoxin into the rice cultivar Minghui 63 (*Oryza sativa* L.) via *Agrobacterium*-mediated genetic transformation, which confers a high level of resistance to leaf-folders and stem-borers [4]. A conventional *indica* cultivar, Minghui 63, was included in this study and is the parental cultivar counterpart to T1C-1. In addition, the conventional *japonica* cultivar Zhonghua11 was also included in this study. Minghui 63 and Zhonghua11 were selected to evaluate metagenomic and metabolomic variation within non-*Bt* plants of different genomic and phenotypic backgrounds. T1C-1 seeds were obtained from the Shanghai Agrobiological Gene Center (Shanghai, China), while Minghui 63 and Zhonghua11 plants were preserved in our laboratory. Plants of the three cultivars were hydroponically grown in International Rice Research Institute (IRRI) rice nutrient solution within a glasshouse at the Shanghai Academy of Agricultural Sciences beginning on May 16, 2018. The aim of the growth experiments was to determine the development stage at which the levels of Bt proteins released from T1C-1 roots were highest. Among all of the growth periods, Bt protein content peaked at the panicle differentiation stage (Supplementary Fig. S1; Supplementary Table S1). Subsequent experiments were conducted within a glasshouse at the Shanghai Academy of Agricultural Sciences (121°33’ E, 31°23’ N) on May 20, 2019 according to local conventional agriculture operations. The experiment consisted of a randomized block design with twelve replicate plots. Paired surface water and soil (rhizosphere and bulk soil) samples collection are described in the Supplemental Material.

### Environmental parameter analyses

Several physicochemical parameters were measured in surface waters. Briefly, pH was measured with a digital pH meter. Nitrate (NO_3_^−^-N) concentrations were determined using UV spectrophotometry [28]. Ammonia (NH_4_^+^-N) and nitrite (NO_2_^−^-N) concentrations were measured using Nessler’s reagent spectrophotometry and the N-(1-naphthyl)-ethylenediamine photometric method, respectively [28]. Total dissolved phosphorus (TDP) and phosphate-P were determined using the ammonium molybdate spectrometric method [29] and the ascorbic acid method [30], respectively. Total dissolved N (TDN) was measured using an Antek 7000 TN Analyzer [31]. Cry1C protein concentrations in surface water samples were determined by enzyme-linked immunosorbent assays as described by Tank [19].

Soil pH was determined by preparing a suspension of air-dried soil sample in water at a ratio of 1:5 (w/v), followed by measuring pH with a digital pH meter (PHS-3C, Shanghai). Organic matter (OM) in soil was determined using the potassium dichromate oxidation method [32] and total nitrogen (TN) with the Kjeldahl procedure [33]. Nitrate (NO_3_^−^-N), ammonium (NH_4_^+^-N), and nitrite (NO_2_^−^-N) concentrations were estimated as described by Cataldu [34], Weatherburn [35], and Steven and Oaks [36], respectively. Soil total P (TP) was determined after digesting with a H_2_SO_4_-HClO_4_ solution at 250 °C and measurement with the molybdenum-blue colorimetric method [37]. Soil Cry1C proteins were quantified with enzyme-linked immunosorbent assays, as described by Dohrmann [6]. All of the measurements were made in triplicate and data are expressed as the averages of the results.

### DNA extraction and metagenomic sequencing

Soil and surface water microbial genomic DNA was extracted in triplicate using an E.Z.N.A.^®^ Soil DNA Kit (Omega Bio-tek, Norcross, GA, U.S.) according to the manufacturer’s protocols. The concentration and purity of extracted DNA was determined with NanoDrop2000 instruments (Thermo Fisher Scientific, Wilmington, DE). DNA extract quality was checked with 1% agarose gel electrophoresis. Purified DNA was then stored at −80 °C for subsequent Illumina HiSeq sequencing as described in Dai [38] on the Hiseq 2500 sequencing platform at Majorbio Bio-Pharm Technology Co., Ltd. (Shanghai, China). Further details for whole-genome shotgun library generation, quality control of sequence reads, and metagenomic profiling analysis are provided in the Supplementary Materials. Metagenomic datasets were deposited in the NCBI Short Read Archive database under SRA accession number SRP276813.

### Collection of root exudates

Roots from the abovementioned plants were gently washed with sterile deionized water three times. Plants were then placed in sterile 800 ml Erlenmeyer flasks and completely submerged in 500 ml of sterile deionized water. Three replicates were included from each cultivar and each replicate comprised ten individually-grown rice plants. Plants were maintained in an incubator at 32 °C for 24 h to collect root exudates. The exudates were then concentrated to 10 ml using a rotary vacuum evaporator (Rotavapor R-100, Buchi Labortechnik AG, Flawil, Switzerland) at 65 °C.

### Metabolite profiling

Rice surface water and root exudates in triplicate were filtered through 0.22 μm polycarbonate membranes (Millipore, USA) prior to the determination of metabolomic profiles. Three replicates per soil samples (1 g) and 200 µl of liquid samples were accurately weighed and metabolites were extracted from them using 400 µl of a methanol:water (4:1, v/v) solution. The mixture was allowed to settle at −20 °C and then processed with a high throughput tissue crusher (Wonbio-96c, Shanghai Wanbo Biotechnology Co., Ltd.) at 50 Hz for 6 min, followed by vortexing for 30 s and ultrasonication at 40 kHz for 30 min at 5 °C. The samples were then maintained at −20 °C for 30 min to precipitate proteins. After centrifugation at 13,000 *g* at 4 °C for 15 min, the supernatant was carefully transferred to sample vials for metabolite profiling using a UPLC-triple-Triple-TOF-MS system (AB SCIEX, Foster City, CA, USA). Sample quality control and metabolomic analyses are described in the Supplementary Materials.

### Growth effects of metabolites on specific microorganisms

*Terrimonas ferruginea* (Beijing YuWei Technology Co., Ltd.) was grown at 30 °C in beef extract agar medium containing peptone (5.0 g/L), beef extract (3.0 g/L), agar (15.0 g/L), and distilled water up to 1.0 L, followed by pH adjustment to 7.0–7.2. Media were sterilized with filtering through 0.22 μm polycarbonate membranes (Millipore, USA). Metabolites (hexylphenyl acetate, Trp-P-1, cinncassiol D2, 20-hydroxy-leukotriene E4) were reconstituted to 100 mM in DMSO before dilution for dosage assays. Overnight bacterial cultures were diluted 100-fold in appropriate media and 10 ml of each were dispensed into Erlenmeyer flasks containing metabolites or DMSO controls. Erlenmeyer flasks were shaken to ensure homogeneity and bacterial growth was monitored by absorbance at 600 nm in a Tecan Infinite M200 pro-microplate reader (Mnnedorf, Switzerland). Values recorded for DMSO controls and metabolite-treated triplicates were averaged. Additional details regarding the growth effects of metabolites on *Runella slithyformis* are provided in the Supplementary Materials.

### Statistical analyses

Data were tested for normality and equal variance. One-way analysis of variance (ANOVA) were performed in the SPSS 19.0 software package (SPSS Institute, Inc., 2010) to determine differences in soils and surface physico-chemical properties, in addition to microbial communities among different plants cultivar treatments. Shortest significant range (SSR) tests were used for multiple post hoc comparisons. Statistically significant differences were defined at *p* < 0.05. Spearman’s rank correlation analysis was performed with the compounds released as root exudates and corresponding community compositional data to evaluate if root exudation influenced soil microbial communities. To understand putative mechanistic associations of microbial community features and metabolites, covariation between representative differentially abundant metabolites and microorganisms or enzymes for different cultivars was evaluated using Spearman’s rank correlation with two-tailed *p* values (*p* < 0.05). Particular attention was paid to those parameters that were FDR-significant.

## Results

### *Bt* rice led to the redistribution of soil nitrogen

To characterize the influence of *Bt* rice on soil environmental biochemistry, samples were first separated into two portions including soils and surface waters. Bt proteins were not detected in surface waters from all cultivars (Supplemental Table S2). However, Bt protein contents for rhizospheres from all three cultivars and bulk soils ranged between 64.14 and 126.68 pg/g soil (Supplemental Table S3). Bt protein contents in samples from *Bt* rice grown in IRRI rice nutrient solution reached 850 pg/ml (Supplementary Table S1). We speculated that the vast majority of Bt protein released from *Bt* plants was bound tightly to soil particles and was thus difficult to isolate, purify, and detect. Total N, NH_4_^+^-N, NO_3_^−^-N, and NO_2_^−^-N contents in T1C-1 rhizospheres were significantly higher than in the Minghui 63 rhizospheres, although the soil pH of T1C-1 rhizospheres was also significantly lower than for Minghui 63 soils (Supplemental Table S3). Interestingly, the total N, NH_4_^+^-N, and NO_3_^−^-N contents in the Zhonghua11 rhizospheres were significantly higher than in the Minghui 63 rhizospheres, pointing to an apparent impact of genotypic differences from different conventional cultivars on soil nitrogen. No differences in organic matter and total P contents were identified among all soil samples (Supplemental Table S3). In addition, the surface waters of T1C-1 exhibited higher NO_3_-N contents than Minghui 63 soils, but lower pH values than Minghui 63 (Supplemental Table 2), consistent with soil results.

### *Bt* rice altered soil microbial communities, but not surface water communities

**Fig. 2:**
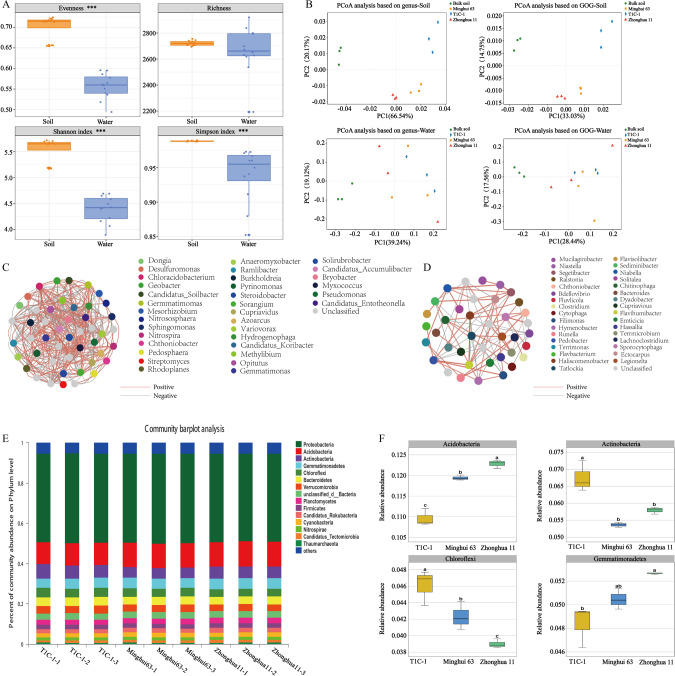
Comparison of soil and surface water shotgun metagenomic sequencing data. **A** Differences in α-diversity metrics, Shannon–Wiener index (*H*′), Simpson index (*D*), Richness (*R*), and Evenness (*E*) between soil and surface water communities. Black asterisks indicate that the α-diversity index was significantly higher in soils (^***^, *p* < 0.001; Wilcoxon rank sum test). **B** Principal coordinates analysis (PCoA) of soil and surface water sample community differences based on Bray–Curtis distances among metagenomic profiles. Co-occurrence networks based on Spearman’s correlation analysis of the abundances for the 50 most abundant microbial genera in soils (**C**) and surface waters (**D**). **E** Relative abundances (%) of the major bacterial phyla present in rhizosphere microbial communities. **F** Bacterial phyla with significantly different (*p* < 0.05) abundances in soils among different rice varieties. Bars with different letters are significantly different (*p* < 0.05).

Soil and surface water samples were collected and analyzed to characterize metagenomic profiles associated with different cultivars. A total of 11,529,157 and 2,880,919 genes were obtained for soil and surface water samples, respectively (Supplementary Table S4). The α diversity indices, Shannon–Wiener index (*H’*), Simpson index (*D*), and Evenness (*E*) were significantly higher in soils than in surface waters, but significant differences were not observed for Richness (*R*) (Fig. 2A). Except for *R*, the α diversity indices *E*, *H*′, and *D* were significantly higher in the T1C-1 rhizosphere than in the other samples, suggesting that *Bt* rice increased soil microbial diversity rather than altering taxonomic compositions. Differences in α diversity indices were not observed among all of the surface water samples (Supplementary Table S5). Principal coordinates analysis (PCoA) (Fig. 2B) based on microbial taxonomic level (genera) and functional classifications (clusters of orthologous groups of proteins, COG) indicated that soil samples from different rice cultivars and bulk soils formed distinct clusters in ordination space. These distinct groupings were not observed for surface water samples, suggesting that *Bt* rice cultivation altered soil microbial community composition and functions, but these changes did not occur in surface waters. The rhizospheres of T1C-1, Minghui 63, and Zhonghua 11 shared substantial overlap in total genera (Supplementary Fig. S2A). In addition, 40 genera specifically inhabited T1C-1 rhizospheres (Supplementary Fig. S2B). To further identify taxa that were differential between T1C-1 and Minghui 63 soils, the 50 most abundant genera that were differentially abundant for T1C-1 or Minghui 63 were specifically analyzed using a *T*-test. Among these, 33 were elevated in T1C-1 soils compared with Minghui 63 soils (Supplementary Fig. S3). Thus, the strongest enrichment was observed for taxa in T1C-1 soils, which is consistent with the general increased α diversity indices for T1C-1 communities (Supplementary Table S5).

Network analysis can provide comprehensive insights into microbial community dynamics and help identify co-occurrence patterns at phylogenetic levels. Co-occurrence network analysis was performed based on the 50 most abundant microbial genera in soils and surface waters. Soil networks consisted of 48 nodes (i.e., genera) and 926 edges (Fig. 2C), while surface water networks comprised 39 nodes and 154 edges (Fig. 2D). The average network transitivity was higher in soil (0.7355) than in surface water (0.4588) networks. In contrast, the average shortest path length of soil samples (1.7243) was lower than for surface water samples (3.6505). These properties suggested that the soil microbial community network was significantly larger and more complex than that of the surface waters. The average modularity and negative correlations in microbial co-occurrence networks were evaluated to assess soil microbiome stability across plant cultivar treatments. Soil microbial networks comprised a larger proportion of negative correlations in T1C-1 soils compared to the Minghui 63 network (Supplementary Fig. S4). The presence of negative interactions contributes to the stability of co-oscillations in communities and promotes the stability of networks. Modular structure analysis indicated that nodes in the networks of T1C-1, Minghui 63, Zhonghua11, and bulk soils grouped into three major modules, respectively, indicating that T1C-1 soils did not exhibit decreased network modularity (Supplementary Fig. S5). *Candidatus Accumulibacter*, *Haliangium*, *Rhodoplanes*, *Variovorax*, *Lysobacter*, *Dongia*, *Ramlibacter*, etc. were identified as keystone taxa defined as highly connected taxa for T1C-1. The most abundant phyla in rhizosphere of different cultivars are shown in Fig. 2E. ANOVA analysis identified four phyla (Actinobacteria, Chloroflexi, Acidobacteria, and Gemmatimonadetes) that were significantly different between different cultivar communities. Actinobacteria and Chloroflexi abundances were highest in T1C-1 rhizospheres compared to soils of other cultivars, while Acidobacteria and Gemmatimonadetes abundances were lowest in T1C-1 rhizospheres (Fig. 2F).

### *Bt* rice cultivation did not significantly alter the abundances of potential probiotic or phytopathogenic microorganisms

**Fig. 3:**
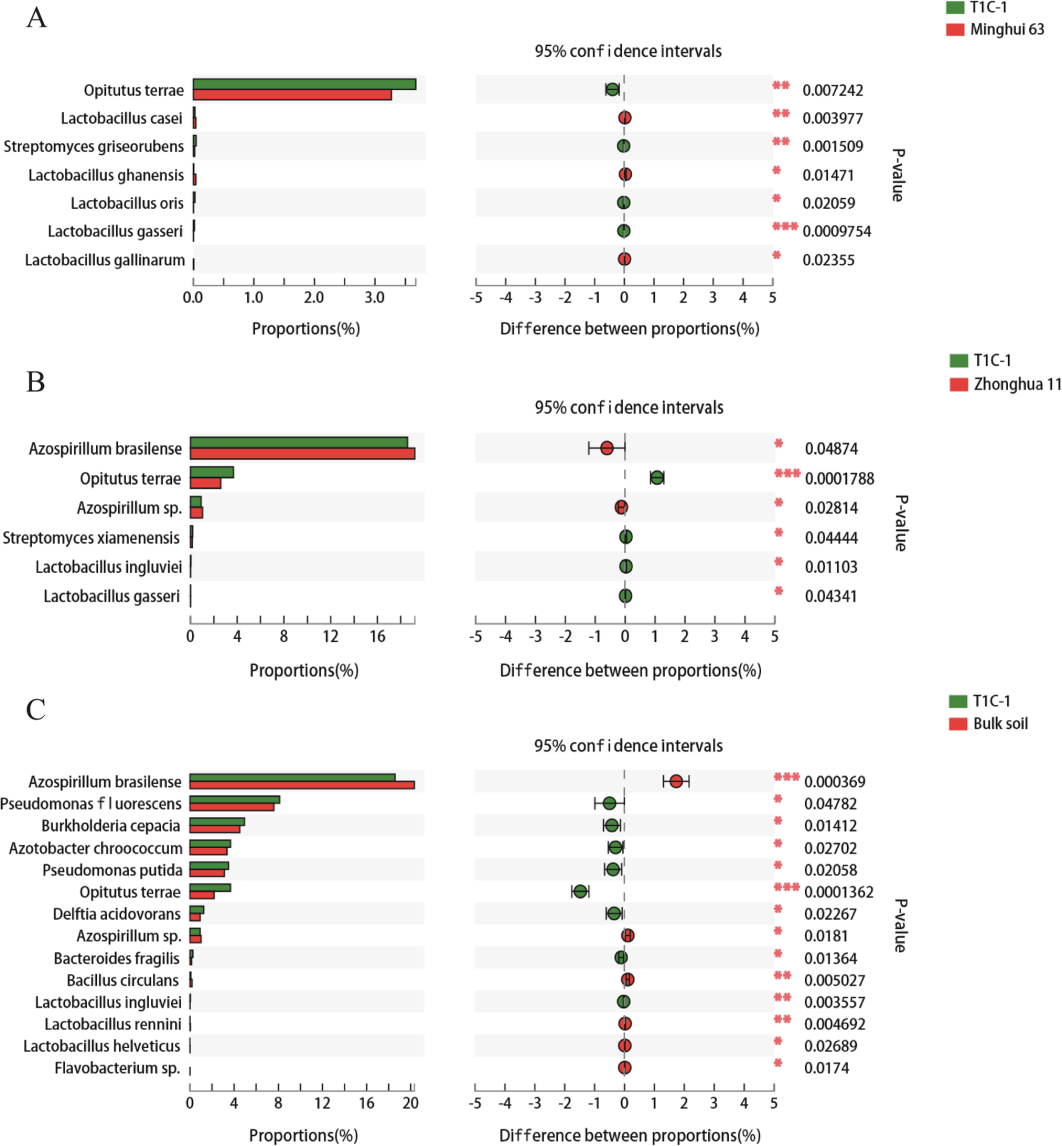
Differentially abundant probiotic microorganisms between paired sample comparisons.

The effects of GM planting on potential probiotic or plant pathogen microbial taxa is an important aspect of environmental risk assessment for GM plants. Of the seven probiotic microorganisms that were differentially abundant in the rhizosphere of T1C-1 and Minghui 63, four exhibited enriched abundances and three exhibited decreased abundances in the T1C-1 rhizosphere compared to those of Minghui 63 (Fig. 3). Notably, the three taxa with decreased abundances belonged to the *Lactobacillus* genus that was also among the enriched microbial taxa. We speculate that the three *Lactobacillus* with decreased abundances did not decrease the beneficial impacts on plants demonstrated by other *Lactobacillus*. When considering paired communities of T1C-1 against Zhonghua 11 or T1C-1 against bulk soils, the proportion of increased or depleted probiotic microorganisms followed similar trends, indicating that T1C-1 plants tended to recruit rhizosphere bacteria that could aid in plant health and fitness. In addition, 21 microbial plant pathogens exhibited significantly different abundances in the rhizospheres of T1C-1 and Minghui 63 (Supplementary Fig. S6). Among all of the differentially abundant microorganisms, 10 exhibited increased abundances and 11 exhibited decreased abundances in the rhizosphere of T1C-1 plants relative to those of Minghui 63 plants. In particular, T1C-1 plants significantly inhibited the growth of the dominant phytopathogenic taxa *Xanthomonas oryzae* compared to Minghui 63 plants. Similar results were observed when comparing the communities of T1C-1 against Zhonghua 11 or T1C-1 against bulk soil communities, suggesting that T1C-1 planting alone altered the identity of the pathogenic microorganisms, but it did not trigger the accumulation of pathogenic microorganisms. Taken together, these results indicated that no adverse effects were apparent on soil microbial communities due to T1C-1 planting.

### Associations between nitrogen-transforming microorganisms with variation in different N forms among *Bt* rice cultivation soils

**Fig. 4:**
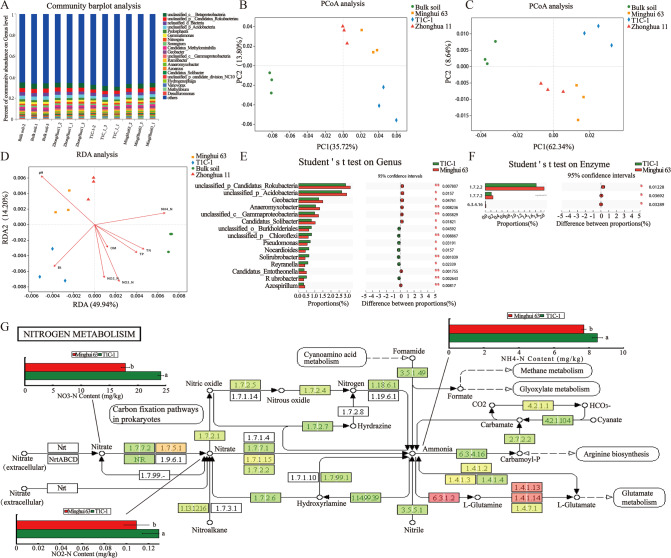
Variation in the soil microbes and their function in nitrogen-transforming. **A** Relative abundances of major microbial genera in soil samples. Principal coordinates analysis (PCoA) based on microbial genus level (**B**) and COG (Clusters of Orthologous Groups) function (**C**) profiles of samples. **D** RDA of soil physico-chemical properties and microbial community compositions at the genus level. Microbial genera (**E**) and enzymes (**F**) that significantly differed between T1C-1 and Minghui 63 rhizosphere metagenomes. **G** Metabolic pathways identified in this study as important distinguishing features based on rhizosphere metagenomic analysis comparison between T1C-1 and Minghui 63 soils. The box in the figure represents a pair of samples, and the depth of color represents the change of enzyme abundance among different samples. Enzyme Commission (EC) Numbers can be inquired in the website: https://www.brenda-enzymes.org/all_enzymes.php.

*Bt* rice cultivation led to altered distributions of N forms in soils and thus, the effects of *Bt* rice on nitrogen-transforming microbial taxa were further investigated. A total of 41,543 non-redundant genes were identified that were involved in nitrogen-transforming reactions among the total 11,529,157 genes present in the soil samples. In addition to unclassified microorganisms, the most abundant microbial genera associated with nitrogen transformations were *Pedosphaera*, *Gemmatimonas*, *Nitrospira*, and *Sorangium* (Fig. 4A). PCoA analysis demonstrated that variation in the abundances of nitrogen-transforming taxa and their associated functions largely separated T1C-1 communities from Minghui 63 communities, consistent with the broad differences observed in soil profiles between these two cultivars (Fig. 4B, C). The relationships between soil properties and nitrogen-transforming microbial genera were also analyzed with RDA (Fig. 4D). The first two RDA components explained 64.14% of the total variation in genera-level composition. Soil pH was most correlated with variation in nitrogen-transforming genera composition, followed closely by NO_3_^-^-N, NO_2_^−^-N and NH_4_^+^-N (Supplementary Table S6). A paired sample *t* test approach was used to identify microbial genera that were significantly enriched or depleted in T1C-1 soils compared to Minghui 63 soils. The 15 most abundant nitrogen-transforming microbial genera with significant differences in abundance between T1C-1 and Minghui 63 soils included *Candidatus Rokubacteria*, unclassified *Acidobacteria*, *Geobacter*, and *Anaeromyxobacter*, among others (Fig. 4E). In addition, three nitrogen-transforming enzymes exhibited significantly different abundances between T1C-1 and Minghui 63 metagenomes including ferredoxin-nitrate reductase [EC:1.7.7.2], nitrite reductase (cytochrome c-552) [EC: 1.7.2.2], and carbamoyl-phosphate synthase (ammonia) [EC:6.3.4.16] (Fig. 4F).

The associations between soil microbial functions and the contents of different N forms were also evaluated. The levels of the aforementioned enzymes were lower in T1C-1 soils relative to Minghui 63 soils and these were particularly prevalent in the nitrogen metabolism pathway (ko 00910) (Fig. 4G). Considering the higher NH_4_-N, NO_3_-N, and NO_2_-N contents in T1C-1 rhizospheres compared with Minghui 63 rhizospheres (Supplementary Table S3 and Fig. 4G), such relationships are consistent with different N forms acting as enzyme substrates. Thus, these results suggest that the cultivation of *Bt* rice altered soil nitrogen transformations by regulating the expression of associated genes. Similar results were also observed when comparing the paired samples of T1C-1 against Zhonghua 11 (Supplementary Fig. S7) or Zhonghua 11 against Minghui 63 (Supplementary Fig. S8).

### Soil metabolomic profiles were altered by *Bt* rice cultivation, but not in surface waters

**Fig. 5:**
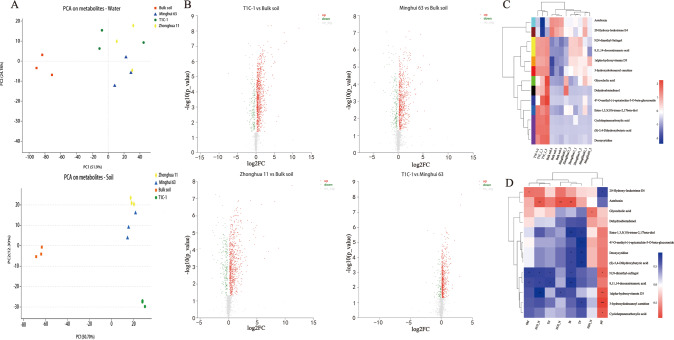
Metabolomic analysis of soil and surface water samples. **A** Principal component analysis (PCA) of soil and surface water metabolomic profiles based on Bray–Curtis distances. **B** Relative abundances of different metabolites. The volcano plot was obtained by plotting the log2 fold change of metabolites on the *x*-axis and the –log10 (*p* value) on the *y*-axis. Metabolites that increased twofold or more with a *p* < 0.05 from paired samples are indicated in red. Metabolites that decreased twofold or more with *p* < 0.05 from a pair samples are indicated in green. The other metabolites are indicated in gray. **C** Heatmap cluster analysis of the 13 representative metabolites in the rhizosphere samples of T1C-1 relative to the other samples. A dendrogram was generated for the samples to show sample differences and is indicated on the left side of the heatmap. Sample similarities were calculated using Bray–Curtis distances. **D** Correlations between representative metabolites and soil physicochemical properties.

Changes in the metabolites that microorganisms may directly or indirectly interact with in soils were subsequently investigated using untargeted metabolomics. These analyses were used to assess the responses of microbial metabolic processes to *Bt* rice cultivation and were guided by the metagenomic insights into taxonomic and functional potentials. A total of 6527 and 6998 metabolites were detected in soils and surface waters, respectively. Among of these, a total of 527 and 539 metabolites were identified in soils and surface waters, respectively. PCA analysis of the metabolite profiles indicated that the first and second principal components significantly separated T1C-1 and Minghui 63 (or Zhonghua 11) metabolic profiles, suggesting that *Bt* rice significantly altered the low molecular weight metabolite profile in soils (Fig. 5A). Similar patterns were not observed in surface water metabolite profiles (Fig. 5A). A total of 988 metabolites were significantly up-regulated and 326 metabolites were significantly down-regulated in T1C-1 rhizospheres compared with Minghui63 rhizospheres (Fig. 5B), further suggesting that T1C-1 planting generally positively impacted soil metabolomes. Moreover, up-regulated metabolites were significantly higher than down-regulated metabolites in plant rhizospheres compared to bulk soils, indicating that plant cultivation increased soil metabolites. Discrimination with VIP values for OPLS-DA model variables led to the identification of 13 representative metabolites among the 200 most abundant metabolites (VIP > 1, *p* < 0.05) that differed in abundance between T1C-1 soils and others (Supplementary Fig. S9). The 13 representative metabolites comprised lipids, nucleotides, organoheterocyclic compounds, organic acids, and other compounds that enabled clear separation of T1C-1 profiles from others (Fig. 5C). Correlations between the 13 representative metabolites and soil physico-chemical properties were further analyzed (Fig. 5D). Soil Bt protein concentrations were positively correlated to the contents of deoxycytidine, N,N-dimethyl-Safingol and (S)-3,4-dihydroxybutyric acid, but negatively correlated to asteltoxin contents.

### Associations between *Bt* rice-linked microorganisms and metabolites

A multi-omic framework integrating soil microbiomes and metabolomes was constructed to identify robust associations in the variation of differentially abundant microorganisms and metabolites in *Bt* and conventional rice variety soils. These robust associations contributed to characterize possible mechanistic relationships that occurred to *Bt* rice. To identify representative bacterial and eukaryotic taxa and metabolites most likely to explain differences among different soil samples, biomarker analysis was conducted with linear discriminant analysis (LDA) effect size (LEfSe) tests (Supplementary Figs. S10–S12). A total of 9017 and 6540 FDR-significant (*q* < 0.05) associations were identified between representative differentially abundant metabolites and bacterial or eukaryotic genera (Supplementary Fig. S13). A total of 24,000 bacterial and 10,750 eukaryotic associations between metabolites and genera were identified, with 37.6% and 60.8% being statistically significant, respectively. Although many metabolites were associated with one or more genera, they tended to not be mechanistically associated with most genera (and vice versa). In addition, a total of 978 associations with T1C-1 were identified, including 967 positive associations and 11 negative correlations that further contributed to understanding the putative mechanistic relationships that are altered when T1C-1 is grown compared to conventional rice.

To understand the functional consequences of microbial community changes due to T1C-1 planting, we first functionally profiled genes in the metagenomes and then summed their abundances according to Enzyme Commission (EC) annotations. The LEfSe approach was again used, but with abundance data of microbial gene encoding enzymes, revealing 27 representative enzymes that were differentially abundant (FDR-corrected *q* < 0.05) in T1C-1, Minghui 63, Zhonghua 11, or bulk soil metagenomes (Supplementary Fig. S14). A total of 1,661 FDR-significant (*q* < 0.05) associations (18.0% of all associations) were observed between representative differentially abundant enzymes and metabolites.

### *Bt* rice re-establishes the carbon metabolism of soil microbial communities

**Fig. 6:**
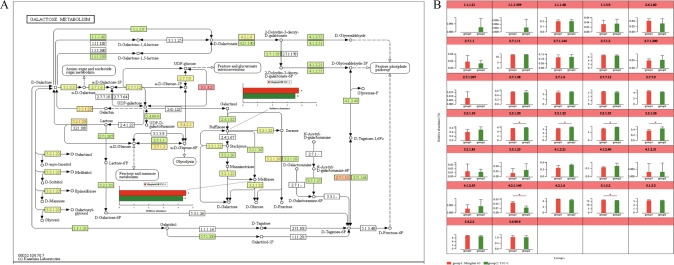
Differentially enriched KEGG pathways revealed by combined ‘omics’ analyses. KEGG enrichment pathways identified as differential between T1C-1 and Minghui 63 communities based on rhizosphere metabolomic (**A**) and metagenomic (**B**) analyses.

A total of 105 metabolites annotated as standards were differential in T1C-1 soils compared with Minghui 63 soils, including 78 up-regulated and 27 down-regulated metabolites (VIP > 1 and *p* < 0.05) (Supplementary Fig. S15). The KEGG pathways associated with representative metabolites were primarily involved in lipid metabolism, carbohydrate metabolism, and energy metabolism, among other areas (Supplementary Fig. S16). Combined ‘omics’ approaches revealed that differentially enriched KEGG pathways in T1C-1 metagenomes compared to Minghui 63 metagenomes included galactose metabolism (ko00052), which is a component of carbohydrate metabolism (Fig. 6). In addition, the abundances of raffinose and melibiose were lower in T1C-1 soils compared to Minghui 63 soils based on non-targeted metabolomic profiling (Fig. 6A). Consistently, the abundances of microbial gene encoding enzymes involved in raffinose and melibiose-related metabolic pathways were significantly higher in T1C-1 metagenomes relative to Minghui 63 metagenomes, including alpha-glucosidase [EC: 3.2.1.20], alpha-galactosidase [EC: 3.2.1.22], and beta-fructofuranosidase [EC: 3.2.1.26] (Fig. 6B). These results suggest a re-establishment of carbon metabolism pathways in which raffinose and melibiose involvement might be attributed to the modulation of associated gene expression.

### Putative mechanisms underlying the correlation of Bt rice-linked root exudate metabolites with rhizosphere microorganisms

A total of 6236 metabolites were detected in rice root exudates, of which 649 metabolites were structurally identified. PCA analysis indicated clear separation of the T1C-1 metabolite profiles from the Minghui 63 and Zhonghua 11 metabolomes, clearly suggesting altered metabolite profiles among different cultivars (Supplementary Fig. S17A). Significantly up-regulated metabolite fold-changes were higher than the significantly down-regulated metabolites in root exudates of T1C-1 profiles compared against Minghui63 or Zhonghua 11 profiles, further suggesting that T1C-1 planting contributed to enrichment of root exudates (Supplementary Fig. S17B–D). Thirty of the most abundant metabolites that were differentially enriched in T1C-1 profiles compared to Minghui 63 profiles were identified, including 27 up-regulated and three down-regulated metabolites (VIP > 1 and *p* < 0.05) (Supplementary Fig. S17E). A total of 81 identical metabolites were identified from root exudates and rhizosphere samples, among which 25 metabolites exhibited similar patterns of variation and highly statistically significant correlations (Spearman’s *r* > 0.7 and *p* < 0.05) between root exudate and rhizosphere profiles (Supplementary Table S7).

**Fig. 7:**
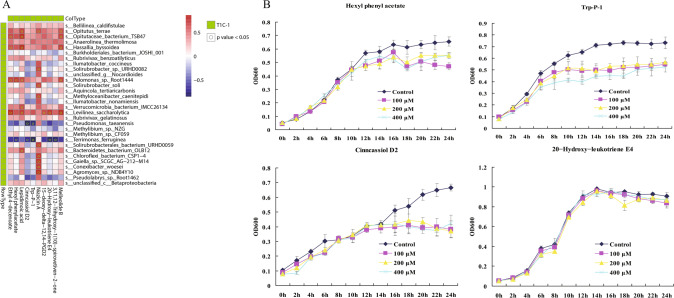
Associations between T1C-1-representative root exudate metabolites and microbial taxa. Associations between representative root exudate metabolites of *Bt* rice and microbial species (**A**) and validation of four predicted metabolite-microorganism relationships (**B**).

To determine how root exudates of different rice cultivars influence soil microbiomes, Spearman’s correlation analysis was conducted using compounds released as root exudates and the rhizosphere microorganisms that exhibited significant differences in abundances in soils of different plants. Acidobacteria abundances were significantly (*p* < 0.05) correlated with most root exudate compounds (194 correlations) while Chloroflexi were correlated with the least amount of compounds (27 correlations) (Supplementary Table S8). To identify whether a specific class of root exudate metabolite was associated with soil microbial communities, root exudates were classified as amino acids, carbohydrates, lipids, and organoheterocyclic compounds. Lipid metabolite levels were significantly correlated with the abundances of Acidobacteria, Actinobacteria, Chloroflexi, and Gemmatimonadetes (166 significant correlations; Supplementary Table S9), followed by organoheterocyclic compounds (54), amino acids (46), and carbohydrates (31). Numerous possible associations between T1C-1-representative root exudate metabolites and microbial taxa were observed (Supplementary Fig. S18, Fig. 7A). To experimentally validate the potential for T1C-1-representative metabolites to exert effects on the growth of T1C-1-representative microbial species, we cultured *Terrimonas ferruginea* that was dominant in soils, in the presence of four metabolites that it was associated with (Fig. 7B). Among the four predicted negative associations, three metabolites (except 20-hydroxy-leukotriene E4) indeed inhibited the growth of *Terrimonas ferruginea* at the tested concentrations. These results provide direct evidence for the influence of *Bt* rice-representative root exudates on *Bt* rice-representative microorganisms.

## Discussion

### Different effects of *Bt* rice on the physiochemical properties of soils and surface waters

GM crops have been rapidly adopted in many countries, becoming the fastest adopted crop technology in the world. The planting area of GM crops with combined traits of insect-resistance and herbicide-tolerance increased by 6% in 2019, and currently represent an estimated 45% of the GM crops in the world [2]. Recent studies have primarily assessed the effects of *Bt* crops on terrestrial environments [8, 39], while the effects on aquatic ecosystems have not been evaluated [40]. Some *Bt* crops, including *Bt* rice are accompanied by a layer of surface water during the development of plants in most growth stages. In addition, the surface waters from croplands along with ditches and underfield drains ultimately drain into rivers or lakes. To evaluate the potential effects of Bt exposure on soils and waters, the response of physico-chemical properties of soils and surface waters to the cultivation of *Bt* rice was investigated here. Bt proteins were not detected in surface waters (Supplemental Table S2) and the concentrations of Bt proteins in soils were significantly lower than in IRRI rice nutrient solutions (Supplemental Table S1; Supplemental Table S3), providing indirect evidence that Bt proteins from root exudates are tightly bound to soil particles and are thus difficult to isolate, purify, and detect [41]. Altogether, these results showed that root exudation did not generally affect the physico-chemical properties of surface waters (Supplemental Table S2).

In contrast, soil physico-chemical properties including total N, NH_4_^+^-N, NO_3_^−^-N, NO_2_^−^-N, and pH exhibited significant differences when comparing T1C-1, Minghui 63, and Zhonghua 11 soils (Supplemental Table S3). However, T1C-1 planting did not consistently impact soil physico-chemical properties compared with results for the other two conventional cultivars. These results contrast with those from a previous study that did not observe significant differences in soil properties when comparing growth of *Bt* and conventional crops [8, 42]. Van Overbeek and Van Elsas [43] reported that microbial community structure was strongly affected by different plants, genotypes (cultivars and lines), plant growth stage, field locations, and experimental duration. Consequently, causal relationships between soil microbial populations and soil properties like different N forms were subsequently characterized by microbial metagenomic analyses in this study.

### Plant root exudates of *Bt* rice alters soil nitrogen by likely influencing soil microbiomes

Several previous studies have used plate counts of cultivable organisms [20] or 16 S ribosomal RNA amplicon sequencing [6, 8] to analyze microbial communities in *Bt* crop soils. In this study, a metagenomic approach was used, revealing the presence of 15,743 soil microbial species that were assigned to bacteria, eukaryota, archaea, and viruses. Thus, this high-throughput analysis allowed the detection of much less abundant members of soil communities by over two orders of magnitude relative to lower-throughput sequencing methods, that are typically limited to the most dominant 100 phylotypes [20]. In addition, ferredoxin-nitrate reductase [EC:1.7.7.2], nitrite reductase (cytochrome c-552) [EC: 1.7.2.2], and carbamoyl-phosphate synthase (ammonia) [EC:6.3.4.16] abundances were differently enriched in T1C-1 and Minghui 63 soils (Fig. 4F, G). Further, the abundances of nitrite reductase (NADH) [EC: 1.7.1.15] and nitrite reductase (cytochrome, ammonia-forming) [EC: 1.7.2.2] were significantly lower in T1C-1 metagenomes relative to Zhonghua 11 metagenomes, while glutamine synthetase [EC: 6.3.1.2] abundances were significantly higher (Supplementary Fig. S7). These results are consistent with differences in soil NO_2_^−^-N and NH_4_^+^-N in T1C-1 and Zhonghua 11 soils. Similar results were also observed when evaluating the association between soil microbial gene encoding enzymes and the contents of different N forms between Zhonghua 11 and Minghui 63 soils (Supplementary Fig. S8). Together, these data demonstrate a likely mutual covariation and causal relationship between soil microbial enzyme abundances and soil nitrogen levels, which was also observed in paired sample comparisons of T1C-1 and Minghui 63 soils. Dohrmann [6] assumed that the degradation of Bt protein released in root exudates from *Bt* plants will degrade into amino acids that ultimately lead to increased ammonia in soils. We previously characterized the proportion of Bt proteins among total protein contents in the rhizospheres of *Bt* rice, revealing low abundances [22]. Hence, changes in soil nitrogen between *Bt* and non-*Bt* rice may not be related to Bt proteins, but rather to small-molecule metabolites in root exudates, as revealed by metabolomic profiles herein. Altogether, these results demonstrate that the effects of *Bt* rice on soil properties, microbial taxa, and microbial gene encoding enzymes were essentially equivalent to effects from conventional *Bt-*lacking rice cultivar.

In this study, we investigated the associations of soil microbial species and microbial gene encoding enzymes involved in nitrogen metabolism, with root exudate metabolites that were differentially abundant in *Bt* rice rhizosphere soils relative to non-*Bt* control plant soils. Three enzymes exhibited lower abundances in T1C-1 soils relative to Minghui 63 soils, including ferredoxin-nitrate reductase [EC:1.7.7.2] and nitrite reductase (cytochrome c-552) [EC: 1.7.2.2]. Examination of microbial species-level functional annotations revealed that communities were not dominated by any single species (Supplementary Fig. S19), suggesting that their enrichment in non-*Bt* controls was better explained by a community-level shift in functional potential, and therefore of greater mechanistic significance [25]. However, carbamoyl-phosphate synthase (ammonia) [EC:6.3.4.16] could be explained by a single microbial species, *Phytophthora sojae*, which largely contributed the enzyme to the community when defining ‘dominant’ as explaining >50% of enzyme copies (Supplementary Fig. S19). Lastly, two dominant microbial species, *Omnitrophica bacterium OLB*16 and *Geothrix fermentans*, exhibited lower abundance in T1C-1 soils compared to Minghui 63 soils and these taxa harbored genes encoding nitrite reductase (cytochrome c-552) [EC: 1.7.2.2] (Supplementary Fig. S19).

**Fig. 8:**
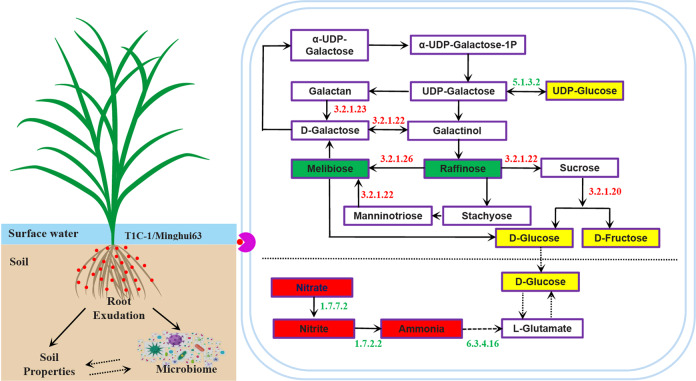
Variation in carbon and nitrogen metabolism due to cultivation of *Bt* rice. Different N forms, metabolites, and enzymes in T1C-1 soils that were significantly increased or depleted compared to Minghui 63 soils are annotated in red or green, respectively.

Integrative analysis of soil microbiomes and root exudate metabolomes revealed 145 root exudate metabolites that differed in abundance between T1C-1 and Minghui 63 soils, and these exudate metabolites were largely associated with *Omnitrophica bacterium* OLB16 and *Geothrix fermentans* (Supplementary Fig. S20A). These observations suggest that root exudate levels may affect changes in the abundances of soil nitrogen-transforming microorganisms associated with *Bt* and non-*Bt* rice varieties. While the results presented here are only correlative, they do highlight that specific components of root exudates from different rice genotypes, including genetically modified genotypes, affect soil microbiomes, especially by altering soil nitrogen levels via the modulation of differently abundant microbial gene encoding enzymes (Fig. 8). Root exudates typically comprise carbon and nitrogen compounds like sugars, amino acids, and carboxylic acids that could directly alter soil physico-chemical properties [44].

### Cultivation of *Bt* rice alters soil metabolomic profiles by affecting galactose metabolism pathways

In this study, we observed that soil metagenomic and metabolomic profiles apparently changed in T1C-1 soils relative to soils of non-*Bt* rice and these results were concomitant with patterns of root exudates for the three cultivars. These results suggest tight couplings of soil metabolomes and microbiomes with root exudates, and the mechanisms underlying this coupling require disentanglement through experimental validation of targeted microbe-metabolite associations [25]. Here, we identified several microbial taxa that contributed alpha-glucosidase [EC: 3.2.1.20], alpha-galactosidase [EC: 3.2.1.22] and beta-fructofuranosidase [EC: 3.2.1.26] enzymes related to galactose metabolism that were differentially abundant in T1C-1 soils relative to Minghui 63 soils (Supplementary Fig. S21). Among these populations, the dominant species *Opitutus terrae*, *Roseiflexus castenholzii*, *Fimbriimonas ginsengisoli*, *Actinobacteria* bacterium. IMCC26207, and *Runella slithyformis* were differentially abundant in T1C-1 soils compared with Minghui 63 soils. These taxa were significantly associated with differentially abundant root exudate metabolites that were identified with multi-omic analyses (Supplementary Fig. S20A). This was especially evident for the positive associations of the *Runella slithyformis* contributed enzyme [EC: 3.2.1.26] with root exudate metabolites, which were higher than the negative associations. The positive associations could be explained by: (1) the metabolite representing a preferred carbon source that promotes species growth, (2) the metabolite occurring as a by-product of species metabolism, or (3) the metabolite selectively inhibiting the growth of other species (or interacting ecologically through another mechanism) [25]. To disentangle the likely scenario among these options and the causal relationships underlying this association of root exudate metabolites and soil microorganisms, monoculture experiments were performed with microbial species in the presence of their associated metabolites. Among the five predicted positive associations, aspirin or pubesenolide were confirmed to enhance *R. slithyformis* growth, while the other predicted associations were not experimentally validated (Supplementary Fig. S20B). Considering confounding factors related to strain specificity, metabolite concentrations, and cultivation times that could impact the results of growth assays, these results provide promising preliminary support for the usefulness of the multi-omic association framework described here for identifying downstream experiments. Altogether, these results reveal that the differential exudates from *Bt* and non-*Bt* rice roots variably influenced the abundances of specific microbiota by modulating gene expression that subsequently resulted in alteration of galactose metabolism pathways (Fig. 8). KEGG pathways that were associated with significantly different exudation metabolites between T1C-1 and Minghui 63 profiles were primarily involved in amino acid, lipid, and nucleic acid metabolism (Supplementary Fig. S22). These results provide support for the experimental verification of unintended alteration of metabolic pathways due to the insertion of exogenous *Bt* genes into plant varieties. Chaparro [27, 45] reported that plant development affected rhizosphere microbiome assemblages due to the alteration of quantity and quality of root exudate phytochemicals at different stages of plant development. Hence, some studies have failed to observe detectable impacts of GM plants on soil microbial communities when considering plants at specific developmental stages [10, 46]. Notably, Bt proteins require cleavage by extracellular proteases into smaller peptides or amino acids before they can enter microbia cells [47]. In addition, Bt proteins are too large to be captured by the LC-MS approach used in this study, but the associations of its derivatives and related compounds with differential root exudates require further investigation.

## Conclusions

This study documents one of the first efforts to identify and validate *Bt* rice-associated changes in soil microbiomes, soil metabolomes, and root exudate metabolomes using an integrated multi-omic framework. *Bt* rice planting can alter soil C-N metabolism by affecting root exudate compositions relative to non-*Bt* rice, in addition to further modulating rhizosphere microbiome assemblages during the growth stage when the highest Bt protein contents are released. Critically, the cultivation of *Bt* rice did not significantly change the abundance of potential probiotic or phytopathogenic taxa and did not reduce soil microbiome stability, suggesting the lack of adverse impacts on environmental agroecosystems by *Bt* rice. Collectively, the results from this study reveal the underlying response mechanisms of soil microbiomes and metabolomes to the cultivation of *Bt* plants, and extend our insights into environmental risk assessments of GM plants.

## Supplementary information

Supplementary material

## Data Availability

Metagenomic datasets were deposited in the NCBI Short Read Archive database under SRA accession number SRP276813.
