# Supplementary material for: Combined metagenomic and metabolomic analyses reveal that Bt rice planting alters soil C-N metabolism
Source: ISME Commun. 2023 Jan 23;3:4. doi: 10.1038/s43705-023-00217-9 (PMC9870860; doi:10.1038/s43705-023-00217-9)
Supplement: Supplementary file 1 — Supplementary material [file 43705_2023_217_MOESM1_ESM.doc]

Supplementary Information

Supplementary Materials, Figures, Tables

for

**Combined metagenomic and metabolomic analyses reveal that *Bt* rice planting alters soil C-N metabolism**

Peng Li^1,2*^, Shuifeng Ye^3^, Jun Chen^4^, Luyao Wang^1^, Yujie Li^1^, Lei Ge^1^, Guogan Wu^1^, Lili Song^1^, Cui Wang^1^, Yu Sun^1^, Jinbin Wang^1^, Aihu Pan^1^, Zhexue Quan^5*^ ,Yunfei Wu^6*^

^1^ Shanghai Key Laboratory of Agricultural Genetics and Breeding, Biotechnology Research Institute, Shanghai Academy of Agricultural Sciences, Shanghai, 201106, China.

^2^ Shanghai Co-Elite Agricultural Sci-Tech (Group) Co., Ltd, Shanghai 201106, China.

^3^ College of Life Sciences, Shangrao Normal University, Shangrao, 334001, China.

^4^ East China University of Technology, Nanchang, 330013, China.

^5^ School of Life Sciences, Fudan University, Shanghai, 200433, China.

### ^6^ The College of Bioscience and Biotechnology, Yangzhou University, Yangzhou, 225009, China.

^*^ Correspondence: pengli13@fudan.edu.cn; [quanzx@fudan.edu.cn](mailto:quanzx@fudan.edu.cn); [006949@yzu.edu.cn](mailto:006949@yzu.edu.cn).

**Supplementary Materials**

**Materials and Methods**

**1. Samples collection and environmental parameter analyses**

*Samples collection*

The experiment consisted of a randomized block design with twelve replicate plots and each plot comprised an area of 1 m × 0.8 m. Soils were classified as Fluvio marine blue purple clay and were thoroughly mixed prior to the experiments. Nine plots contained plants of all three cultivars (one cultivar of each plot) that were planted at a distance of 15 cm between plants. The other three plots without plants were considered controls (i.e., only containing bulk soils) and were subjected to the same agronomic conditions as the above-mentioned nine plots. Paired surface water and soil (rhizosphere and bulk soil) samples were collected at the panicle differentiation stage on August 16, 2019. For surface water sampling, water was collected in a 5 L sterile PET bottle and immediately transported to laboratory at a temperature of 0-4°C. A portion of the water sample was filtered through 0.22 μm polycarbonate membranes (Millipore, USA) to capture microbial cells. The filtered membranes were maintained at -80°C until subsequent DNA extraction (Liu et al., 2018). The remainder was stored at 4°C prior to analysis of water properties. When the surface water was drained, soils within 2 mm of the root surfaces were considered rhizosphere soils (DeAngelis et al., 2009). Plants were gently removed from soils and rhizospheres were collected by gently shaking roots to dislodge small adhering soil clumps. The plants were then used for subsequent collection of root exudates. To ensure representativeness of rhizosphere samples, each sample comprised a composite from three plants of the same cultivar. Samples were placed on ice in a cooler and transported to the laboratory on the same day. Soils were then passed through a 2.0 mm sieve and a portion of the soil sample (collected in triplicate for each cultivar) was stored at -80°C for later DNA extraction, while the remainder was stored at 4°C for analysis of soil physico-chemical properties (Li et al., 2018).

**2. Metagenomic profiling analysis**

*Metagenomic sequencing*

DNA extract was fragmented to an average size of about 400 bp using Covaris M220 (Gene Company Limited, China) for paired-end library construction. Paired-end library was constructed by NEXTFLEX® Rapid DNA-Seq (Bioo Scientific, Austin, TX, USA). Adapters containing the full complement of sequencing primer hybridization sites were ligated to the blunt-end of fragments. Paired-end sequencing was performed on Illumina NovaSeq (Illumina Inc., San Diego, CA, USA) at Majorbio Bio-Pharm Technology Co., Ltd. (Shanghai, China) using NovaSeq Reagent Kits according to the manufacturer’s instructions (www.illumina.com). Sequence data associated with this project have been deposited in the NCBI Short Read Archive database (Accession Number: SRP276813).

*Sequence quality control and genome assembly*

The data were analyzed on the free online platform of Majorbio Cloud Platform (www.majorbio.com). The paired-end Illumina reads were trimmed of adaptors, and low-quality reads (length<50 bp or with a quality value <20 or having N bases) were removed by fastp (Chen et al., 2018) (https://github.com/OpenGene/fastp, version 0.20.0). Metagenomics data were assembled using MEGAHIT (Li et al., 2015) (<https://github.com/voutcn/megahit>, version 1.1.2), which makes use of succinct de Bruijn graphs. Contigs with the length being or over 300 bp were selected as the final assembling result, and then the contigs were used for further gene prediction and annotation.

To estimate α-diversity, the Shannon–Wiener index *H’*, Simpson index *D*, Richness *R*, and Evenness *E* indices were calculated using the mothur software package (v 1.35.1). β-diversity estimates (Bray–Curtis and Hellinger distances) were calculated using the QIIME pipeline (Caporaso et al., 2010). Hierarchical cluster analysis of samples based on Hellinger distances was performed using the R software package (http://www.rproject.org/). RDA was conducted to determine the environmental variables that were most related to the differences in microbial community composition, and the results were used to construct a soil property matrix for variation partitioning analysis in R (v.2.8.1) using a Mantel test with the Pearson correlation method and 1,000 permutations through the “vegan” package (v.1.15-1) (R Core Team, 2006). Potential probiotic and phytopathogenic microbes were identified according to a database of Probiotics Functions and Lineages (http://bidd2.nus.edu.sg/probio/homepage.htm) and a database of Pathogen Host Interactions (PHI) (http://www.phi-base.org/index.jsp).

*Gene prediction, taxonomy, and functional annotation*

Open reading frames (ORFs) from each assembled contig were predicted by using MetaGene (Noguchi et al., 2006) (http://metagene.cb.k.u-tokyo.ac.jp/). The predicted ORFs with length being or over 100 bp were retrieved and translated into amino acid sequences using the NCBI translation table ([http://www.ncbi.nlm.nih.gov/Taxonomy/taxonomyhome.html/index.cgi?chapter=tgencodes#SG1](http://www.ncbi.nlm.nih.gov/Taxonomy/taxonomyhome.html/index.cgi?chapter=tgencodes#SG1). Construction of non redundant gene catalogue using CD-HIT (Fu et al., 2012) (http://www.bioinformatics.org/cd-hit/, version 4.6.1) with 90% sequence identity and 90% coverage. Reads after quality control were mapped to the non-redundant gene catalog with 95% identity using SOAPaligner (Li et al., 2008) (http://soap.genomics.org.cn/, version 2.21), and gene abundance in each sample was evaluated.

[Representative sequences](http://en.wikipedia.org/wiki/Representative_sequences) of non-redundant gene catalog were aligned to NCBI NR database with e-value cutoff of 1e-5 using Diamond (Buchfink et al., 2015) (http://www.diamondsearch.org/index.php, version 0.8.35) for taxonomic annotations. Cluster of orthologous groups of proteins (COG) annotation for the [representative sequences](http://en.wikipedia.org/wiki/Representative_sequences) was performed using Diamond^[7]^ (http://www.diamondsearch.org/index.php, version 0.8.35) against eggNOG database with an e-value cutoff of 1e-5. The KEGG annotation was conducted by Diamond (Buchfink et al., 2015) (http://www.diamondsearch.org/index.php, version 0.8.35) against the Kyoto Encyclopedia of Genes and Genomes database (http://www.genome.jp/keeg/) with an e-value cutoff of 1e-5.

[*Analysis of microbial co-occurrence patterns*](http://www.baidu.com/link?url=OUUnAKp6gkFO0Rg4K0jMGvpvYdMgIh-cx08rUwxPG6bcalzq_F48pV_Ev1B9zbM_F75HRbWaOCIlJd96Iq-nBEpIGaLHNYuumeBr8qzu7FZdvNq_3WKdm3tm6-PN6tRT-Inbvv20WU63_NTIuTt4pm1c_cbN5yFcN_XdAPlZXAWUL7IQrhb6dxpTOLKofA4gwqeVmzuCE7isV3ZsfcJQYygfrLe1FXtZaWa_xw6dJ1lOAFme_2gFovXfaTzlHxlN1htvqyd8sBMXRFw8jao9PgJQrSwkDuB1y_Vfy5EPhiI-WKUtEM0yVJJ9ct2jb7Va&wd=&eqid=db58f510000663bd000000055f3e8949)

Network was used to explore co-occurrence patterns of microbial taxa of soil and surface water. The top 50 most abundant genera (≥ 97% of the total number of microbial genera observed) were selected. A Spearman's correlation between two genera was considered statistically robust if the Spearman's correlation coefficient (ρ) was >0.6 and the *P*-value was <0.05 (Barberán et al., 2012; Jiao et al., 2016). All the robust correlations determined in pairwise comparison of the genera abundance form a correlation network where each node represents one genus, and each edge stands for a strong and significant correlation between the nodes. To describe the topology of the resulting networks, a set of measures (number of nodes and edges, average path length, network diameter, average degree, graph density, clustering coefficient and modularity) was calculated using igraph (Csardi and Nepusz, 2006) packages in R environment and networks were visualized using the interactive platform Gephi (Newman, 2003, 2006; Bastian et al., 2009). Meanwhile, 10,000 Erdös - Rényi random networks were generated to compare with the topology of real network, with each edge having the same probability of being assigned to any node (Erdös and Rényi, 1960). The betweenness centrality of a node reflected the amount of control that this node exerts over the interactions of other nodes in the network. Genera with the highest betweenness centrality value were considered as keystone species (González et al., 2010; Vick-Majors et al., 2014).

*Identification of representative features*

To detect the representative features (bacteria, fungi and enzymes) that were differently abundant in the three varieties, linear discriminant analysis (LDA) effect size (lefse) method based on normalized relative abundance matrix was used.. LEfSe method uses the Kruskal-Wallis test to identify features with significant differences between rhizosphere and control groups and performs LDA to evaluate the effect size of each feature (Segata et al., 2011). An LDA threshold score of 2.0 and *P* < 0.05 were used to detect representative features.

**3. Metabolomic analysis**

*Sample Quality control*

As a part of the system conditioning and quality control process, a pooled quality control sample (QC) was prepared by mixing equal volumes of all samples. The QC samples were disposed and tested in the same manner as the analytic samples. It helped to represent the whole sample set, which would be injected at regular intervals in order to monitor the stability of the analysis.

*UPLC-MS/MS analysis*

Chromatographic separation of the metabolites was performed on a ExionLC^TM^AD system (AB Sciex, USA) equipped with an ACQUITY UPLC BEH C18 column (100 mm × 2.1 mm i.d., 1.7 µm; Waters, Milford,USA). The mobile phases consisted of 0.1% formic acid in water with formic acid (0.1%) (solvent A) and 0.1% formic acid in acetonitrile:isopropanol (1:1, v/v)(solvent B). The solvent gradient changed according to the following conditions: from 0 to 3 min, 95% (A): 5% (B) to 80% (A): 20% (B); from 3 to 9 min, 80% (A): 20% (B) to 5% (A): 95% (B); from 9 to 13 min, 5% (A): 95% (B) to 5% (A): 95% (B) ; from 13 to 13.1 min, 5% (A): 95% (B) to 95% (A): 5% (B), from 13.1 to 16 min, 95% (A): 5% (B) to 95% (A): 5% (B) for equilibrating the systems. The sample injection volume was 20 uL and the flow rate was set to 0.4 mL/min. The column temperature was maintained at 40^o^C. During the period of analysis, all these samples were stored at 4^o^C.

The UPLC system was coupled to a quadrupole-time-of-flight mass spectrometer (Triple TOF^TM^5600+, AB Sciex, USA) equipped with an electrospray ionization (ESI) source operating in positive mode and negative mode. The optimal conditions were set as followed: source temperature, 500^o^C ; curtain gas (CUR), 30 psi; both Ion Source GS1 and GS2, 50 psi; ion-spray voltage floating (ISVF),-4000V in negative mode and 5000V in positive mode, respectively; declustering potential, 80V; a collision energy(CE), 20-60V rolling for MS/MS. Data acquisition was performed with the Data Dependent Acquisition (DDA) mode. The detection was carried out over a mass range of 50-1000 m/z.

*Data preprocessing and annotation*

After UPLC-TOF/MS analyses, the raw data were imported into the Progenesis QI 2.3 (Nonlinear Dynamics, Waters, USA) for peak detection and alignment. The preprocessing results generated a data matrix that consisted of the retention time (RT), mass-to-charge ratio (m/z) values, and peak intensity. Metabolic features detected at least 80% in any set of samples were retained. After filtering, minimum metabolite values were imputed for specific samples in which the metabolite levels fell below the lower limit of quantitation and each Metabolic feature were normalized by sum. The internal standard was used for data QC (reproducibility), Metabolic features which the relative standard deviation (RSD) of QC>30% were discarded. Following normalization procedures and imputation, statistical analysis was performed on log transformed data to identify significant differences in metabolite levels between comparable groups. Mass spectra of these metabolic features were identified by using the accurate mass , MS/MS fragments spectra and isotope ratio difference with searching in reliable biochemical databases as Human metabolome database (HMDB) (http://www.hmdb.ca/) and Metlin database (https://metlin.scripps.edu/). Concretely, The mass tolerance between the measured m/z values and the exact mass of the components of interest was ±10 ppm. For metabolites having MS/MS confirmation, only the ones with MS/MS fragments score above 30 were considered as confidently identified. Otherwise, metabolites had only tentative assignments.

*Multivariate statistical analysis*

A multivariate statistical analysis was performed using ropls (Version1.6.2, http://bioconductor.org/packages/release/bioc/html/ropls.html) R package from Bioconductor on Majorbio Cloud Platform (https://cloud.majorbio.com). Principle component analysis (PCA) using an unsupervised method was applied to obtain an overview of the metabolic data, general clustering, trends, or outliers were visualized . All of the metabolite variables were scaled to unit-variances prior to conducting the PCA. Orthogonal partial least squares discriminate analysis (OPLS-DA) was used for statistical analysis to determine global metabolic changes between comparable groups. All of the metabolite variables were scaled to pareto Scaling prior to conducting the OPLS-DA. The model validity was evaluated from model parameters R2 and Q2, which provide information for the interpretability and predictability, respectively, of the model and avoid the risk of over-fitting. Variable importance in the projection (VIP) was calculated in OPLS-DA model. *p* values were estimated with paired Student’s t-test on Single dimensional statistical analysis.

*Differential metabolites analysis*

Statistically significant among groups were selected with VIP value more than 1 and p value less than 0.05. Differential metabolites among two groups were summarized , and mapped into their biochemical pathways through metabolic enrichment and pathway analysis based on database search (KEGG, http://www. genome.jp/kegg/). These metabolites can be classified according to the pathways they involved or the functions they performed. Enrichment analysis was usually to analyze a group of metabolites in a functional node whether they appear or not. The principle was that the annotation analysis of a single metabolite develops into an annotation analysis of a group of metabolites. scipy.stats (Python packages) ( https://docs.scipy.org/doc/scipy/ ) was exploited to identify statistically significantly enriched pathway using Fisher’s exact test.

**4. Growth effects of metabolites on *Runella slithyformis***

### We grew *Runella slithyformis* ([Beijing YuWei Technology Co., Ltd](http://www.baidu.com/link?url=2nI-C2g0kM2tTqnpoRE2YIRpk5vutdbxg8QOj_LOzSJDwdmmBbsCwjyu8WUZYWSiyHXY5TZuibMDlQAuhH0bh_&wd=&eqid=8d6e6c530011eaf2000000045f3f822d)) in beef extract agar medium at 26 °C. Adjust pH to 6.8-7.2. We sterilized the media using a 0.22-μm polycarbonate membranes (Millipore, USA). All metabolite (Pubesenolidel, Digoxin, permetin A, Aspirin and Pyroglutamic acid) were brought to 100 mM in DMSO before dilution for dose assays. Overnight bacterial cultures were diluted 100-fold in appropriate media and 10 ml were dispensed per Erlenmeyer flasks containing metabolites or DMSO control. The Erlenmeyer flasks were shaken to ensure homogeneity; bacterial growth was monitored (absorbance at 600 nm) in a Tecan Infinite M200 pro-microplate reader (Mnnedorf, Switzerland). The values recorded for DMSO controls and metabolite-treated triplicates were averaged.

**References**

Barberán A, Bates ST, Casamayor EO, Fierer N, 2012. Using network analysis to explore co-occurrence patterns in soil microbial communities. ISME J. 6, 343-351.

Bastian, M., Heymann, S., Jacomy, M., 2009. Gephi: an open source software for exploring and manipulating networks. ICWSM 8, 361-362.

Buchfink B, Xie C, Huson DH. Fast and sensitive protein alignment using DIAMOND. Nat Methods. 2015; 12 (1): 59-60.

Caporaso JG, Kuczynski J, Stombaugh J, Bittinger K, Bushman FD. 2010. QIIME allows analysis of high-throughput community sequencing data. Nat Methods 7: 335–336.

Chen S, Zhou Y, Chen Y, Gu J. fastp: an ultra-fast all-in-one FASTQ preprocessor. Bioinformatics. 2018; 34(17): i884-i890.

Csardi, G., Nepusz, T., 2006. The igraph software package for complex network research. Inter J., Complex Syst. 1695, 1-9.

DeAngelis KM, Brodie EL, DeSantis TZ, Andersen GL, Lindow SE, and Firestone MK. Selective progressive response of soil microbial community to wild oat roots. ISME J. 2009;3,168–178.

Erdös P, Rényi A, 1960. On the evolution of random graphs. Publ. Math. Inst. Hung. Acad. Sci. 5, 17-61.

Fu L, Niu B, Zhu Z, Wu S, Li W. CD-HIT: accelerated for clustering the next-generation sequencing data. Bioinformatics. 2012; 28 (23): 3150-3152.

González, A.M.M., Dalsgaard, B., Olesen, J.M., 2010. Centrality measures and the importance of generalist species in pollination networks. Ecol. Complex. 7, 36-43.

Jiao S, Liu ZS, Lin YB, Yang J, Chen WM, Wei GH. Bacterial communities in oil contaminated soils: biogeography and cooccurrence patterns. Soil Biology Biochemistry 98 (2016) 64-73.

Li D, Liu CM, Luo R, Sadakane K, Lam TW. MEGAHIT: an ultra-fast single-node solution for large and complex metagenomics assembly via succinct de Bruijn graph. Bioinformatics. 2015; 31 (10): 1674-1676.

Li P, Ye SF, Liu H, Pan AH, Ming F, Tang XM. Cultivation of drought-tolerant and insect-resistant rice affects soil bacterial, but not fungal, abundances and community structures. Frontiers in Microbiology. 2018;9:1390.

Li R, Li Y, Kristiansen K, Wang J. SOAP: short oligonucleotide alignment program. Bioinformatics. 2008; 24 (5): 713-714.

Liu T, Zhang AN, Wang JW, Liu SF, Jiang XT, Dang CY, Ma T, Liu ST, Chen Q, Xie SG, Zhang T, Ni JR. Integrated biogeography of planktonic and sedimentary bacterial communities in the Yangtze River. Microbiome. 2018;6:16.

Newman, M.E., 2003. The structure and function of complex networks. Siam Rev. 45, 167-256.

Newman, M.E., 2006. Modularity and community structure in networks. Proc. Natl. Acad. Sci. 103, 8577-8582.

Noguchi H, Park J, Takagi T. MetaGene: prokaryotic gene finding from environmental genome shotgun sequences. Nucleic Acids Res. 2006; 34 (19): 5623-5630.

Segata, N., Izard, J., Waldron, L., Gevers, D., Miropolsky, L., Garrett, W.S., Huttenhower, C., 2011. Metagenomic biomarker discovery and explanation. Genome Biol. 12, R60.

Vick-Majors, T.J., Priscu, J.C., Amaral-Zettler, L.A., 2014. Modular community structure suggests metabolic plasticity during the transition to polar night in icecovered Antarctic lakes. ISME J. 8, 778-789.

Supplemental Table S1. Bt protein content (pg/ml) of each plant root exudates collected and calculated at different growth stages of the rice varieties

| Varieties | Seedling stage | Tillering stage | Panicle differentiation stage | Filling stage |
| --- | --- | --- | --- | --- |
| T1C-1  Minghui 63  Zhonghua 11 | 497.85±47.50^c^  ND  ND | 754.00±52.19^b^  ND  ND | 850.00±54.78^a^  ND  ND | 383.00±10.44^d^  ND  ND |

Different lowercase letters in a line indicate significant difference at 0.05 level; Abbreviation: ND, not detected.

Supplemental Table S2. Physical-chemical characteristics of the surface water samples used in this study^a^

| Sample | TDN (mg/L) | TDP (mg/L) | NH_4_^+^-N (μg/L) | NO_3_^-^-N (μg/L) | NO_2_^-^-N (μg/L) | PO_4_^3-^-P (mg/L) | pH | Bt protein (pg/kg) |
| --- | --- | --- | --- | --- | --- | --- | --- | --- |
| T1C-1  Minghui63  Zhonghua11  Bulk soil | 1.11±0.08 ^a^  1.09±0.11 ^a^  1.08±0.15 ^a^  1.20±0.05 ^a^ | 1.32±0.17 ^a^  1.33±0.07 ^a^  1.34±0.06 ^a^  1.46±0.14 ^a^ | 49.72±8.17 ^b^  43.95±12.78 ^b^  64.56±10.94 ^a^  57.42±7.98 ^ab^ | 21.12±3.36 ^a^  12.44±2.22 ^b^  14.59±2.40 ^b^  17.99±1.56 ^ab^ | 1.23±0.20 ^a^  1.54±0.13 ^a^  1.38±0.21 ^a^  1.34±0.13 ^a^ | 1.09±0.07 ^a^  1.17±0.13 ^a^  1.15±0.05 ^a^  1.16±0.05 ^a^ | 7.38±0.07 ^b^  7.80±0.06 ^a^  7.73±0.06 ^a^  7.26±0.04 ^c^ | ND  ND  ND  ND |

^a^ Values represent the averages of three repetitions and the standard deviations. Different lowercase letters in the same column indicate a statistically significant difference at 0.05 level. Abbreviation: ND, not detected.

Supplemental Table S3. Physicochemical properties of soil samples used in this study^a^

| Treatment | Organic matter (OM) (g/kg) | Total N (TN) (g/kg) | Total P (TP)  (g/kg) | NH_4_-N  (mg/kg) | NO_3_-N  (mg/kg) | NO_2_-N  (mg/kg) | pH | Bt protein (pg/kg) |
| --- | --- | --- | --- | --- | --- | --- | --- | --- |
| T1C-1  Minghui63  Zhonghua11  Bulk soil | 21.35±0.08 ^a^  20.88±0.46 ^a^  21.28±0.27 ^a^  21.06±0.22 ^a^ | 1.31±0.01 ^a^  1.28±0.01 ^b^  1.30±0.01 ^a^  1.32±0.01 ^a^ | 0.89±0.01 ^a^  0.88±0.02 ^a^  0.88±0.01 ^a^  0.91±0.02 ^a^ | 8.48±0.30 ^b^  7.70±0.15 ^c^  10.72±0.38 ^a^  11.14±0.97 ^a^ | 24.02±0.50 ^a^  17.79±0.75 ^b^  21.32±0.41 ^a^  23.40±1.21 ^a^ | 0.13±0.01 ^a^  0.11±0.01 ^b^  0.10±0.00 ^b^  0.13±0.01 ^a^ | 7.77±0.19 ^b^  8.30±0.06 ^a^  7.49±0.04 ^c^  7.45±0.09 ^c^ | 126.68±10.76 ^a^  64.14±3.13 ^b^  67.23±7.66 ^b^  64.57±6.13 ^b^ |

^a^ Values represent the averages of three replicate measurements and the standard deviations. Different lowercase letters in the same column indicate a statistically significant difference at the *p* < 0.05 level.

Supplemental Table S4. Summary of metagenomic sequencing parameters of all samples

| Samples | Raw reads | Raw base (bp) | Clean reads | Clean base(bp) | Percent in raw reads (%) | Percent in raw bases (%) | Total genes |
| --- | --- | --- | --- | --- | --- | --- | --- |
| T1C-1-1-S  T1C-1 -2-S  T1C-1 -3-S  Minghui63-1-S  Minghui63-2-S  Minghui63-3-S  Zhonghua11-1-S  Zhonghua11-2-S  Zhonghua11-3-S  Bulk soil-1-S  Bulk soil-2-S  Bulk soil-3-S  T1C-1 -1-W  T1C-1 -2-W  T1C-1 -3-W  Minghui63-1-W  Minghui63-2-W  Minghui63-3-W  Zhonghua11-1-W  Zhonghua11-2-W  Zhonghua11-3-W  Bulk soil-1-W  Bulk soil-2-W  Bulk soil-3-W | 42238516  44231918  45471602  42928298  45211342  41833530  51046314  46714650  47657794  45332080  43026882  42099912  49211906  49467390  48723094  47812280  46540834  48363934  46883272  49994850  53434990  56777070  48902788  52065264 | 6378015916  6679019618  6866211902  6482172998  6826912642  6316863030  7707993414  7053912150  7196326894  6845144080  6497059182  6357086712  7430997806  7469575890  7357187194  7219654280  7027665934  7302954034  7079374072  7549222350  8068683490  8573337570  7384320988  7861854864 | 41886740  43886528  44982048  42582022  44785444  41389236  50521442  46227200  47117148  44612156  42539150  41696194  48886408  49057377  48377546  47476155  46208025  48092632  46584143  49636552  53064037  56438666  48637788  51743093 | 6315292352  6618053992  6781674816  6422462708  6753358644  6239614747  7618891113  6970704866  7104030611  6714674990  6410302545  6288705502  7346870811  7355694571  7265183736  7131096872  6940750489  7229005131  6998679342  7445290857  7968812818  8490484825  7318815674  7780885751 | 99.16716771  99.21913854  98.92338519  99.19336192  99.05798417  98.93794762  98.97177297  98.95653719  98.86556646  98.41188845  98.86644819  99.04104788  99.33857876  99.17114487  99.29079217  99.29699023  99.28490968  99.43904067  99.36197073  99.28333018  99.30578634  99.4039777  99.45810861  99.381217 | 99.0165662  99.08720696  98.76879585  99.0788538  98.92258768  98.77711005  98.84402728  98.82040941  98.71745288  98.09399059  98.66467836  98.92433102  98.867891  98.47539779  98.7494751  98.77338437  98.76323881  98.98741109  98.86014315  98.62328213  98.76224328  99.03359987  99.11291351  98.97010166 | Soil:  11529157  Surface water:  2880919 |

Abbreviation: S, Soil samples. W, Surface water samples.

Supplemental Table S5. The richness (*R*), Shannon-Wiener index (*H′*), Simpson index (*D*), and evenness (*E*) of microbial communities from soils and surface waters of different rice variety plants ^a^

| Samples | *R* | Soil  *H′* | *D* | *E* | *R* | Surface  *H′* | water  *D* | *E* |
| --- | --- | --- | --- | --- | --- | --- | --- | --- |
| T1C-1  Minghui63  Zhonghua11  Bulk soil | 2719.33±21.96 ^a^  2714.33±11.93 ^a^  2747.00±8.18 ^a^  2706.33±4.51 ^a^ | 5.7086±0.0112 ^a^  5.6622±0.0083 ^b^  5.6469±0.0018 ^c^  5.1835±0.0067 ^d^ | 0.9894±0.0002 ^a^  0.9886±0.0001 ^b^  0.9881±0.0000 ^c^  0.9877±0.0001 ^d^ | 0.7219±0.0021 ^a^  0.7162±0.0007 ^b^  0.7132±0.0005 ^c^  0.6559±0.0008 ^d^ | 2761.00±97.77 ^a^  2521.33±284.66 ^a^  2782.66±147.76 ^a^  2595.33±50.93 ^a^ | 4.6109±0.1058 ^a^  4.3341±0.1505 ^a^  4.5570±0.1488 ^a^  4.0600±0.1642 ^b^ | 0.9703±0.0033 ^a^  0.9460±0.0123 ^a^  0.9609±0.0115 ^a^  0.8810±0.0296 ^b^ | 0.5820±0.0155 ^a^  0.5536±0.0111 ^a^  0.5747±0.0225 ^a^  0.5164±0.0211 ^b^ |

^a^ Values represent the averages of three replicate measurements and the standard deviations. Different lowercase letters in the same column indicate a statistically significant difference at the *p* < 0.05 level.

Supplemental Table S6. Correlation of environmental variables and the first two RDA components.

|  | RDA1 | RDA2 | R^2^ | *P* Value |
| --- | --- | --- | --- | --- |
| OM  TN  TP  NH_4_^+^-N  NO3^-^-N  NO2^-^-N  pH  Bt | 0.38648  0.82521  0.73928  0.97487  0.30593  0.13554  -0.62575  -0.57193 | -0.9223  -0.56483  -0.6734  0.22275  -0.95205  -0.99077  0.78002  -0.8203 | 0.13524  0.43688  0.57362  0.64614  0.83623  0.65805  0.91401  0.61811 | 0.519  0.077  0.012  0.006  0.003  0.006  0.001  0.019 |

Supplemental Table S7. 81 identical metabolites existed in both root exudation and rhizosphere. 25 metabolites marked in red showed similar patterns of variation and highly statistically significant correlation in root exudation and rhizosphere.

| Metabolites |
| --- |
| P-CHLOROPHENYLALANINE  Muramic acid  Trans-EKODE-(E)-Ib  4-Hydroxyretinoic acid  9-OxoODE  N-stearoyl valine  L-Tryptophan  Arginyl-Asparagine  3-Methyl-1-phenyl-1-butanone  Sphingosine  Dihydrodeoxy-8-epiaustdiol  Butyl salicylate  Alpha-CEHC  Trp-P-1  2,3-Butanediol glucoside  3-Hydroxydodecanedioic acid  Tetradecanedioic acid  2-Octenedioic acid  4-Nitrophenol  Myricetin 3-neohesperidoside  Ribothymidine  Hydroxyisonobilin  (±)9-HpODE  2,4-DINITROPHENOL  Cyclocalopin D  13Z-Docosenamide  KAPA  Eicosanoyl-EA  PE(15:0/20:2(11Z,14Z))  C17 Sphinganine  1-Hexanol  Cysteinyl-Glutamine  Genipic acid  20-Hydroxy-leukotriene E4  Riesling acetal  1-Heptanol  Ethylparaben  (S)-10,16-Dihydroxyhexadecanoic acid  LysoPC(18:0)  6-Tridecynoic acid  Methenamine  Melleolide  Kudzusaponin SA4  Corchorifatty acid F  Glucosylsphingosine  Lysyl-Valine  Sphinganine  8,13-dihydroxy-9,11-octadecadienoic acid  C16 Sphingosine  C16 Sphinganine  Triphenylphosphine oxide  Undecanedioic acid  Melleolide B  3,6,7-Trihydroxy-4'-methoxyflavone 7-rhamnoside  Triphenyl phosphate  2,4-Hexadienyl propionate  L-Phenylalanine  Urocanic acid  N-Methyl-1-deoxynojirimycin  2-Carboxy-4-dodecanolide  Hexadecanedioic acid  (2E)-3-(2,4-dihydroxy-5-methoxyphenyl)prop-2-enoic acid  Suberic acid  Aminofructose 6-phosphate  2-Hydroxy-2,6,6-trimethylcyclohexanone  4-Hydroxybenzaldehyde  9-Oxo-nonanoic acid  Dihydrocumambrin A  LysoPC(18:2(9Z,12Z))  2-Dodecylbenzenesulfonic acid  2-hydroxyhexadecanoic acid  Cincassiol B  N-Undecylbenzenesulfonic acid  12-Oxo-20-hydroxy-leukotriene B4  Neocnidilide  1,11-Undecanedicarboxylic acid  Zedoarol  6''-Malonylcosmosiin  Isoaustin  Asteltoxin  (3'x,5'a,9'x,10'b)-O-(3-Hydroxy-6-oxo-7-drimen-11-yl)umbelliferone |

Supplemental Table S8. Spearman’s correlation analysis of the four bacterial phyla significantly associated with compounds released as root exudates.

| Phylum | Number of positive correlations | Number of negative correlations | Total number of correlations |
| --- | --- | --- | --- |
| Actinobacteria  Chloroflexi Acidobacteria Gemmatimonadetes | 54  7  26  20 | 13  20  168  110 | 67  27  194  130 |

The values indicate the number of significant (*p* < 0.05) Spearman’s correlations for each phylum.

Supplemental Table S9. Spearman’s correlation analysis of the four bacterial phyla significantly associated with groups of compounds released as root exudates.

| Phylum | Amino acids  Positively Negatively | Carbohydrates  Positively Negatively | Lipids  Positively Negatively | Organoheterocyclic compounds  Positively Negatively |
| --- | --- | --- | --- | --- |
| Acidobacteria  Actinobacteria  Chloroflexi Gemmatimonadetes  Total Correlations | 8 2  4 14  9 2  6 1  46 | 4 4  4 7  7 0  3 2  31 | 38 4  48 2  31 12  22 9  166 | 10 4  5 12  12 0  6 5  54 |

The values indicate the number of significant (*p* < 0.05) Spearman’s correlations.


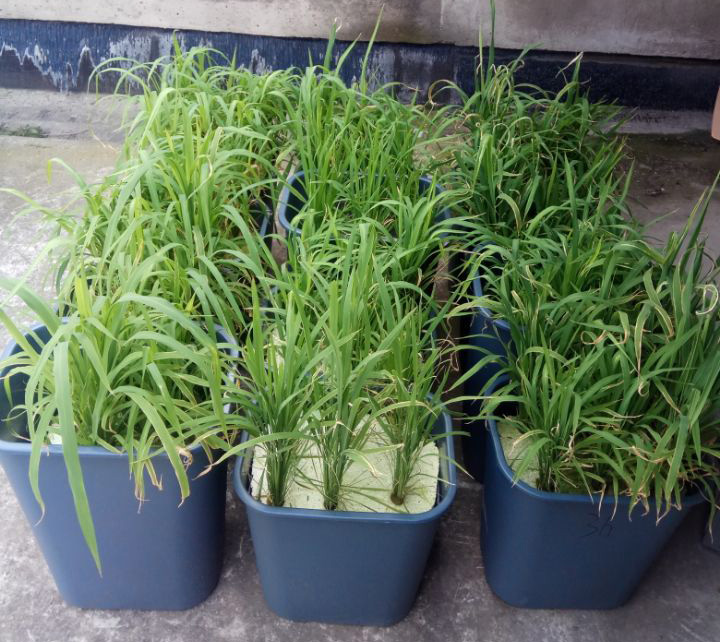


Supplemental Figure S1. Rice grown in IRRI rice nutrient solution at panicle differentiation stages.


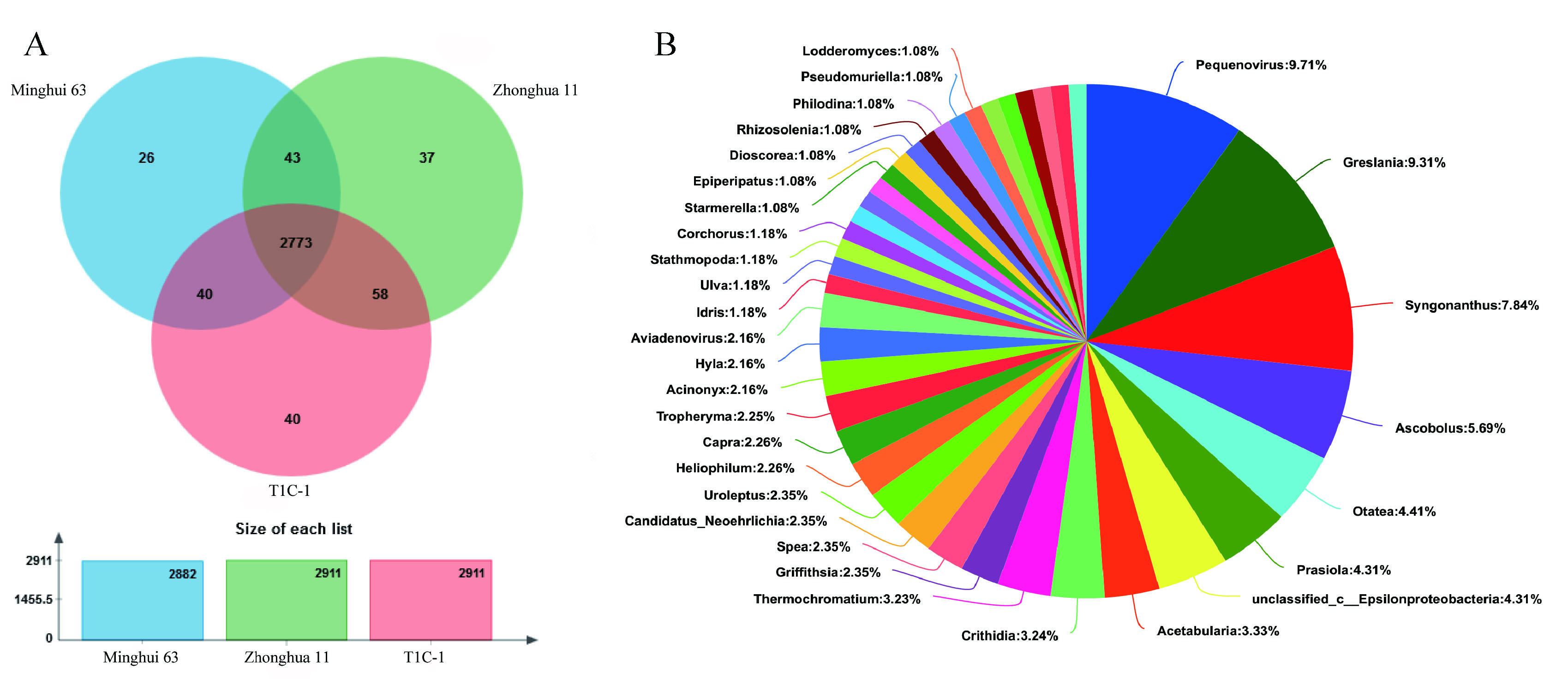


Supplemental Figure S2. Venn diagrams (A) shows substantial overlap of genera in the root microbiota of T1C-1, Minghui 63 and Zhonaghua 11, and pieplot (B) represents the composition for the genera specifically inhabiting T1C-1.


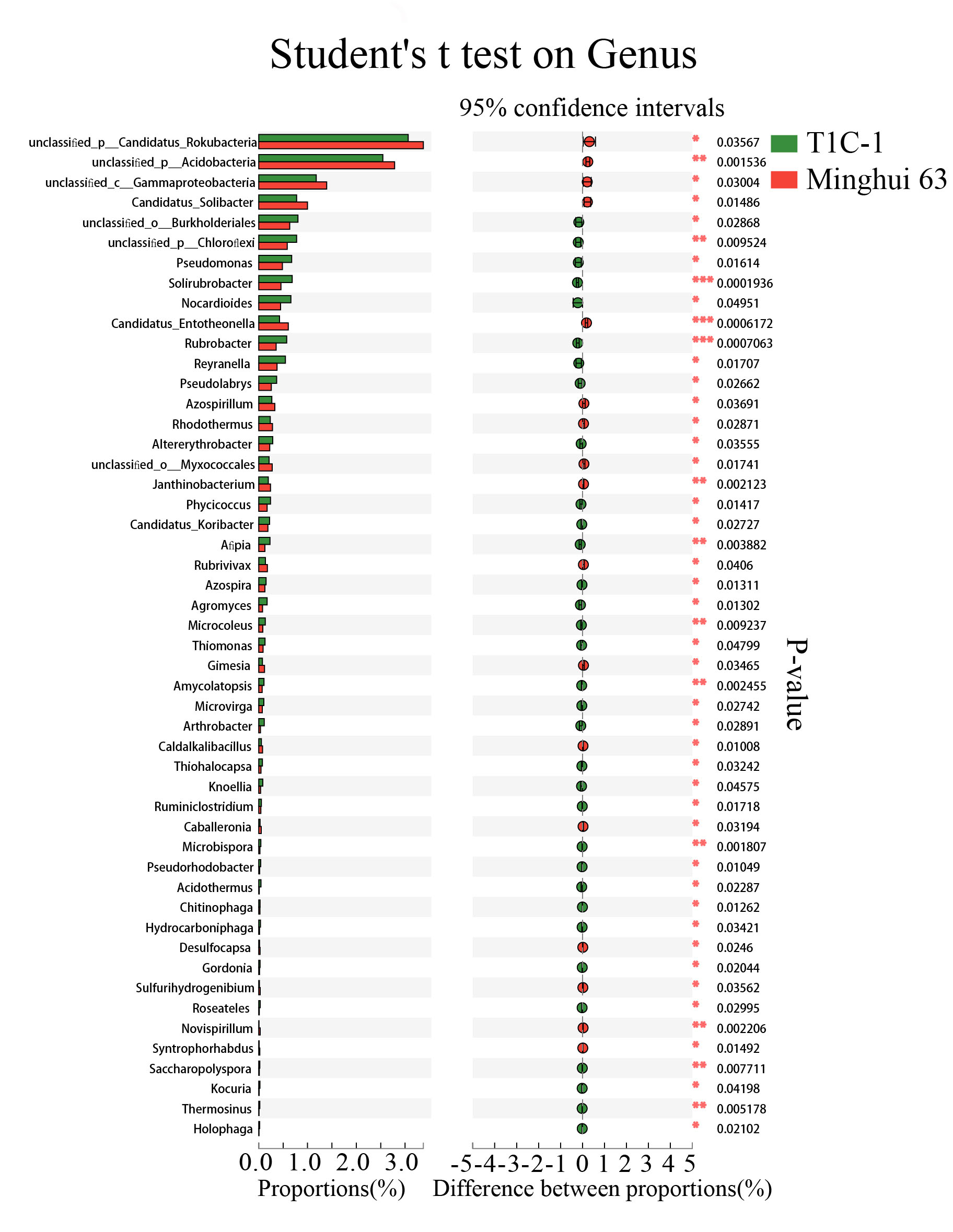


Supplementary Figure S3. Top 50 Differentially abundant genera in T1C-1 or Minghui 63.

**
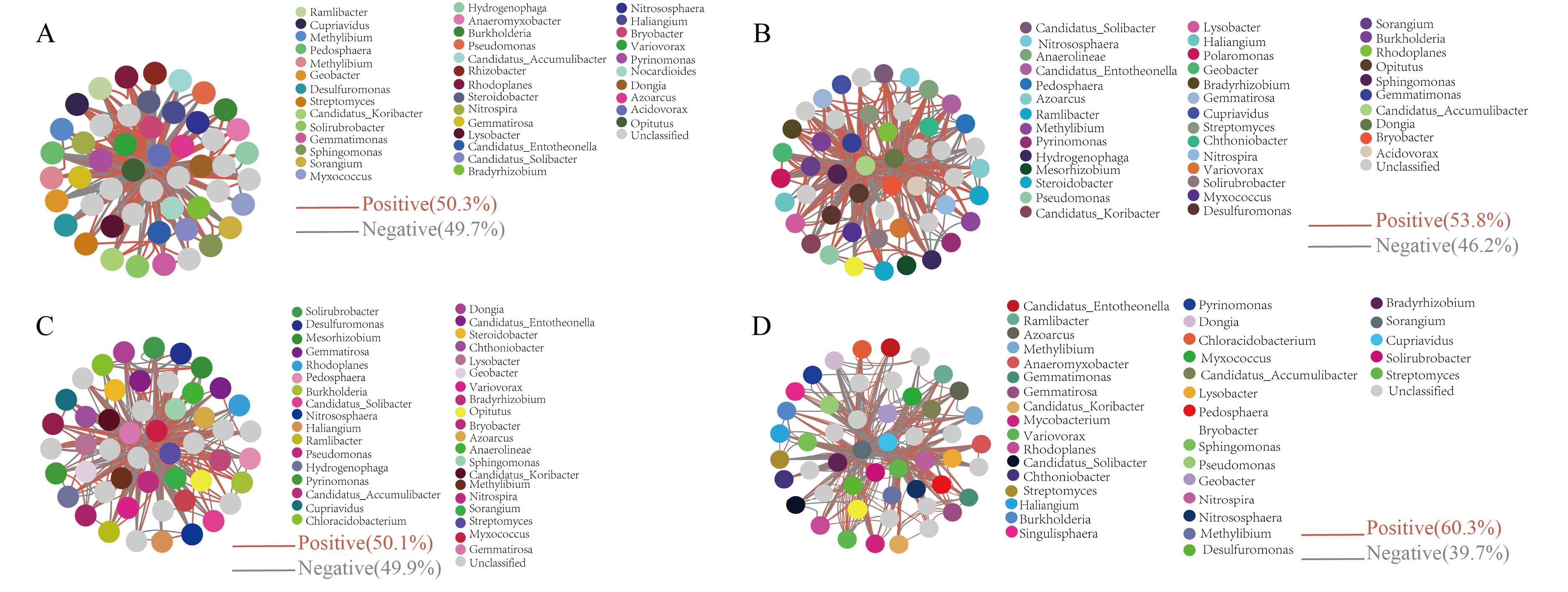
**

Supplementary Figure S4. The co-occurrence network structure of soil microbial community for T1C-1 (A), Minghui 63 (B), Zhonghua 11 (C) and Bulk soil (D) at genera level.

**
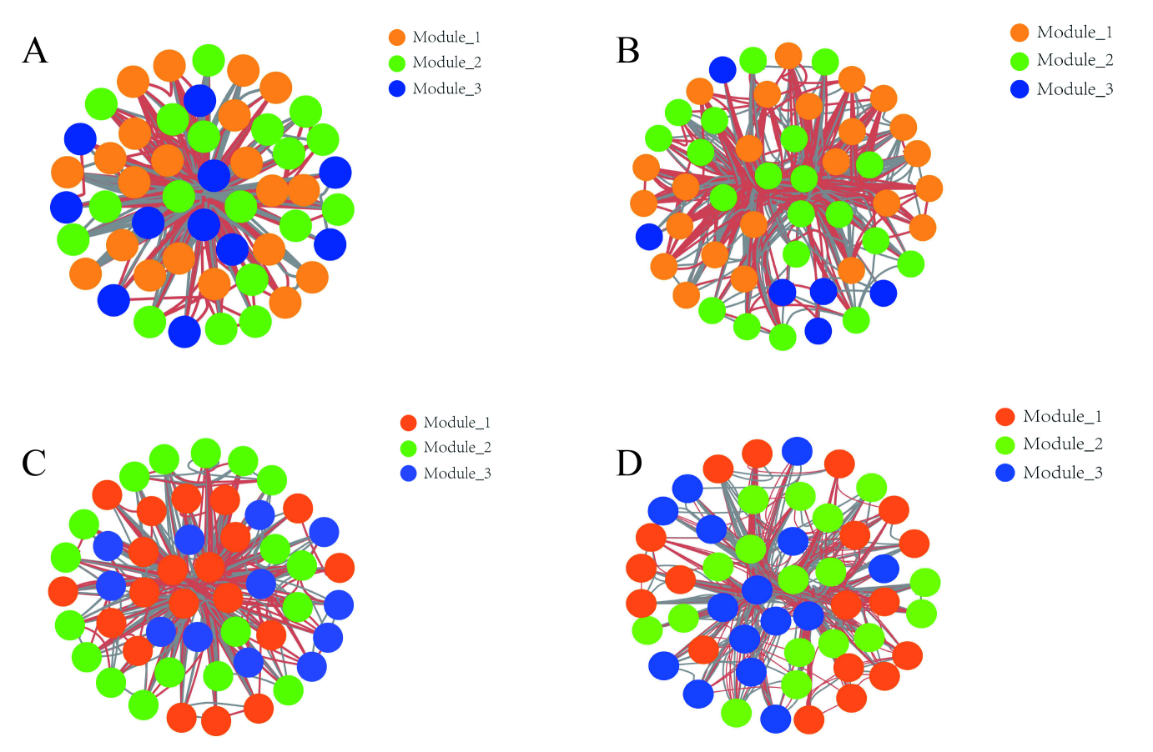
**

Supplementary Figure S5. The co-occurrence network colored by modularity class based on microbial phyla (T1C-1 (A), Minghui 63 (B), Zhonghua 11 (C) and Bulk soil (D)). The thickness of each connection between two nodes (edge) is proportional to the value of Spearman's correlation coefficients.





Supplementary Figure S6. Differentially abundant microbial plant pathogens in paired samples.


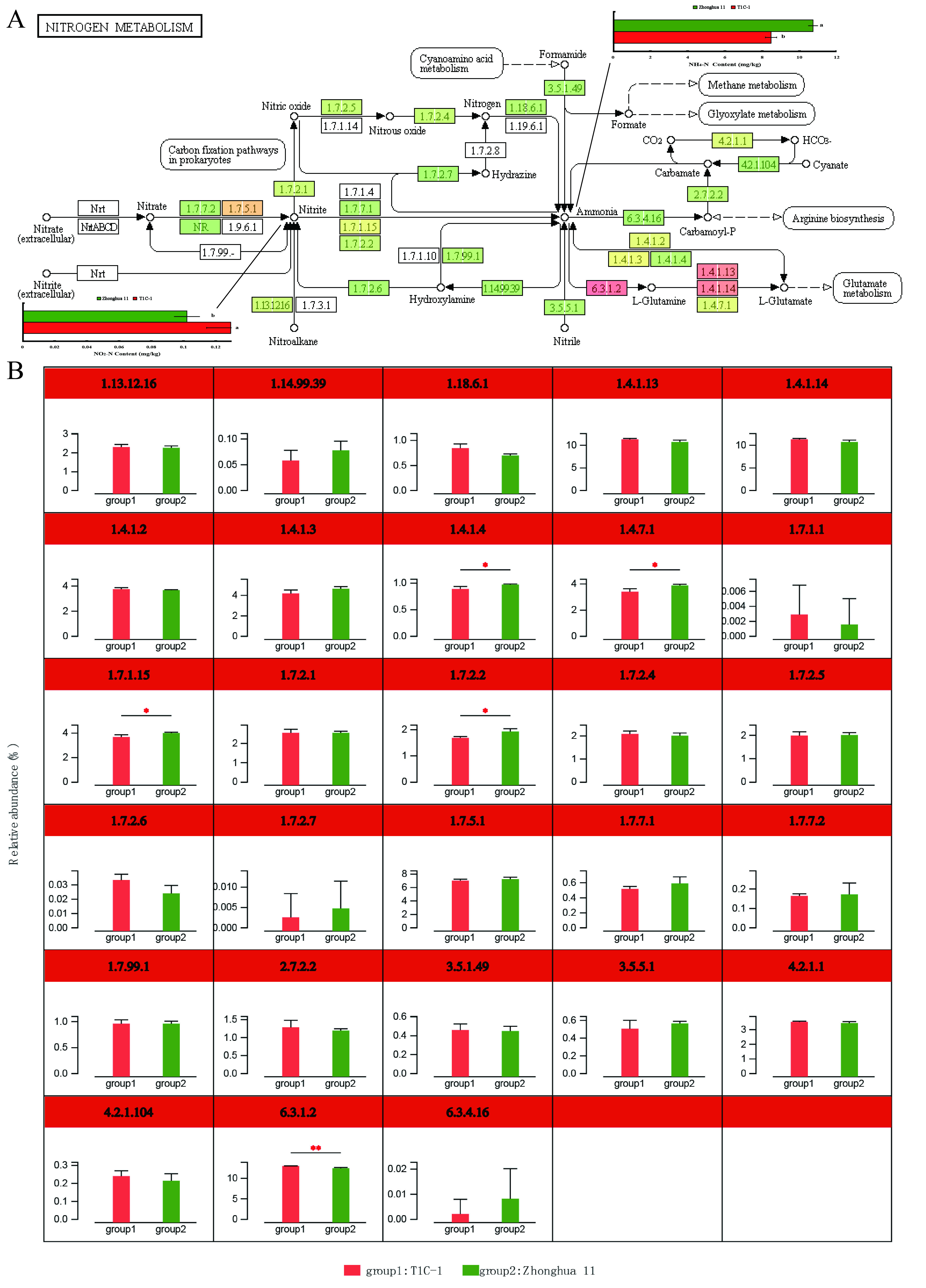


Supplementary Figure S7. Metabolic pathways in this study based on rhizosphere metagenomic analysis between T1C-1 and Zhonghua 11. (A) Nitrogen metabolism pathway (ko 00910) based on the KEGG database; (B) The levels of the microbial enzyme involved in nitrogen metabolism.


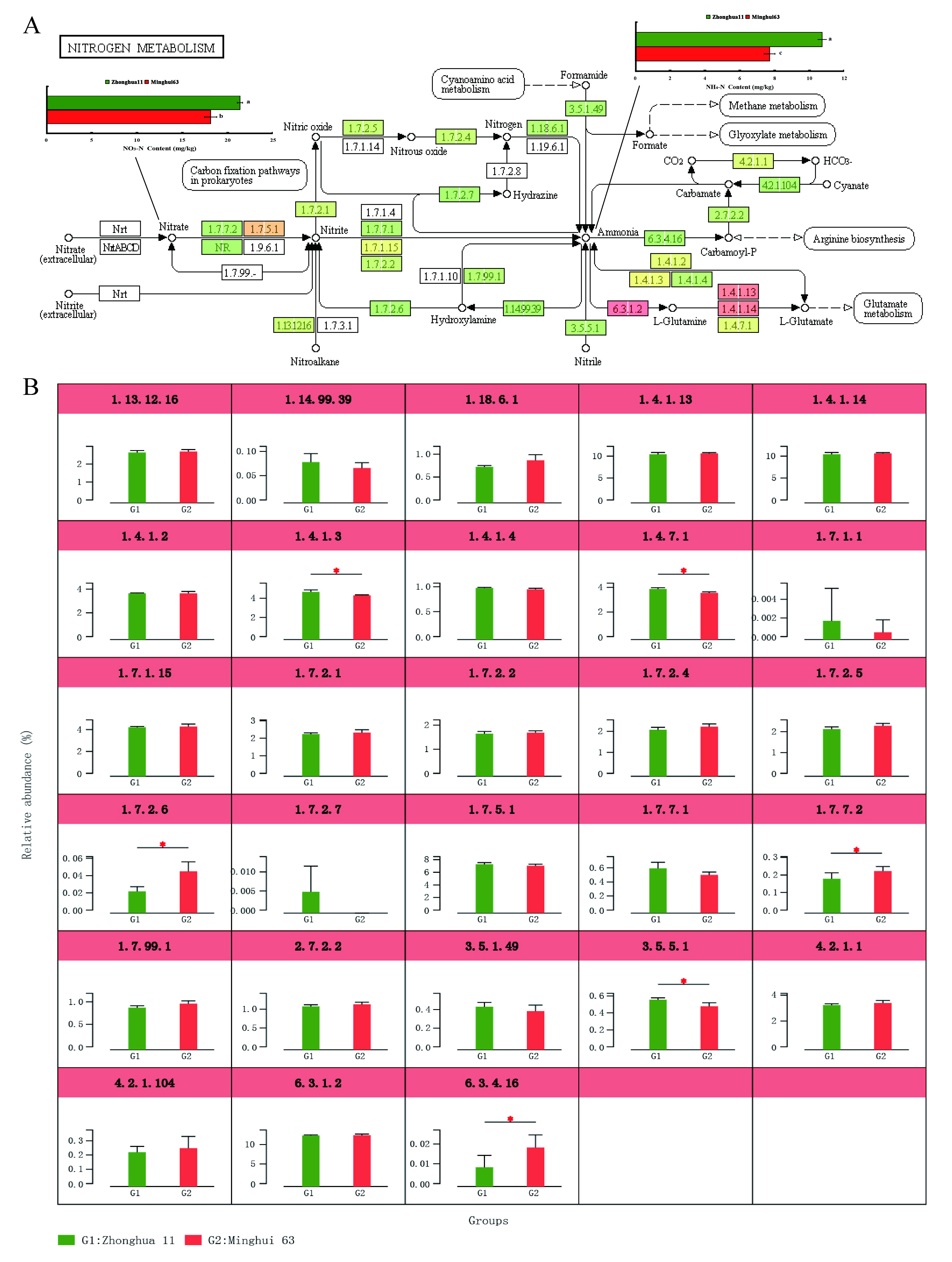


Supplementary Figure S8. Metabolic pathways in this study based on rhizosphere metagenomic analysis between Zhonghua 11 and Minghui 63. (A) Nitrogen metabolism pathway (ko 00910) based on the KEGG database; (B) The levels of the microbial enzyme involved in nitrogen metabolism.


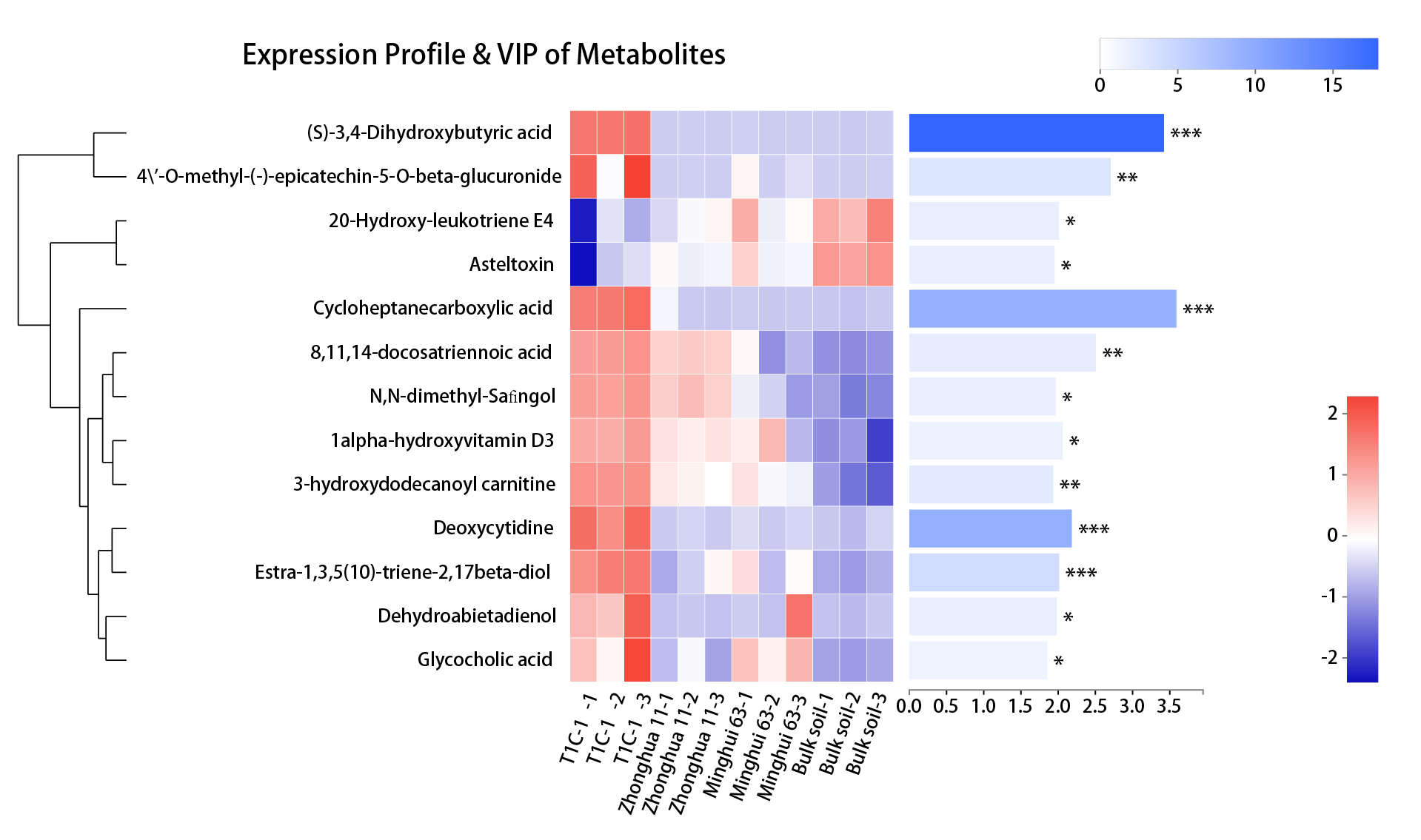


Supplementary Figure S9. Differentially abundant metabolites between T1C-1 and others samples based on the VIP in the OPLS-DA model variable (VIP≥1, *P*<0.05).


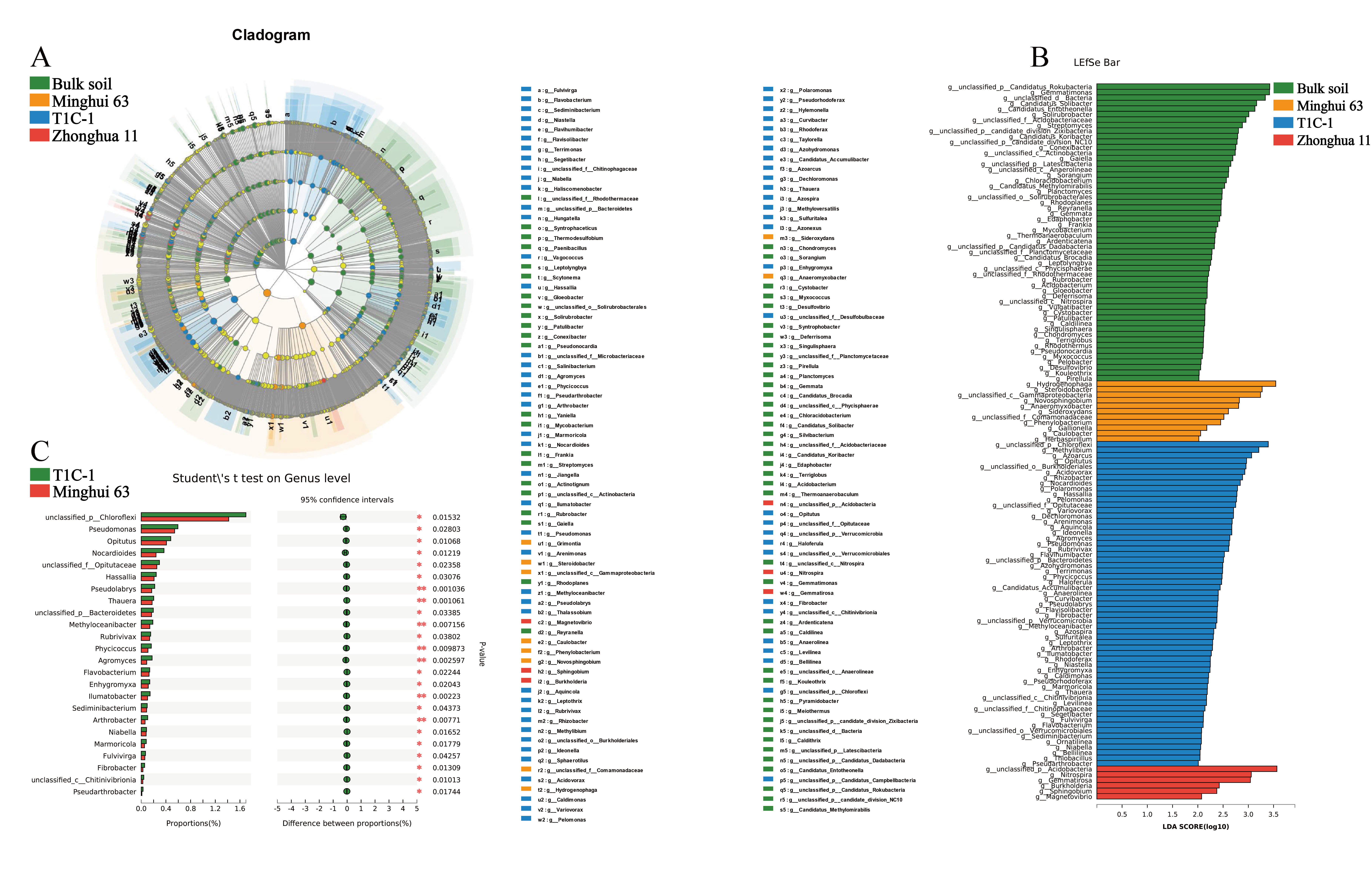


Supplementary Figure S10. LEfSe analysis of soil bacterial abundance. (A) Cladogram of soil bacterial communities. (B) LDA score identified the size of differentiation among three rice varieties and bulk soil control with a threshold value of 2.0. (C) Comparisons of bacterial abundances with significant differences between T1C-1 and Minghui 63.


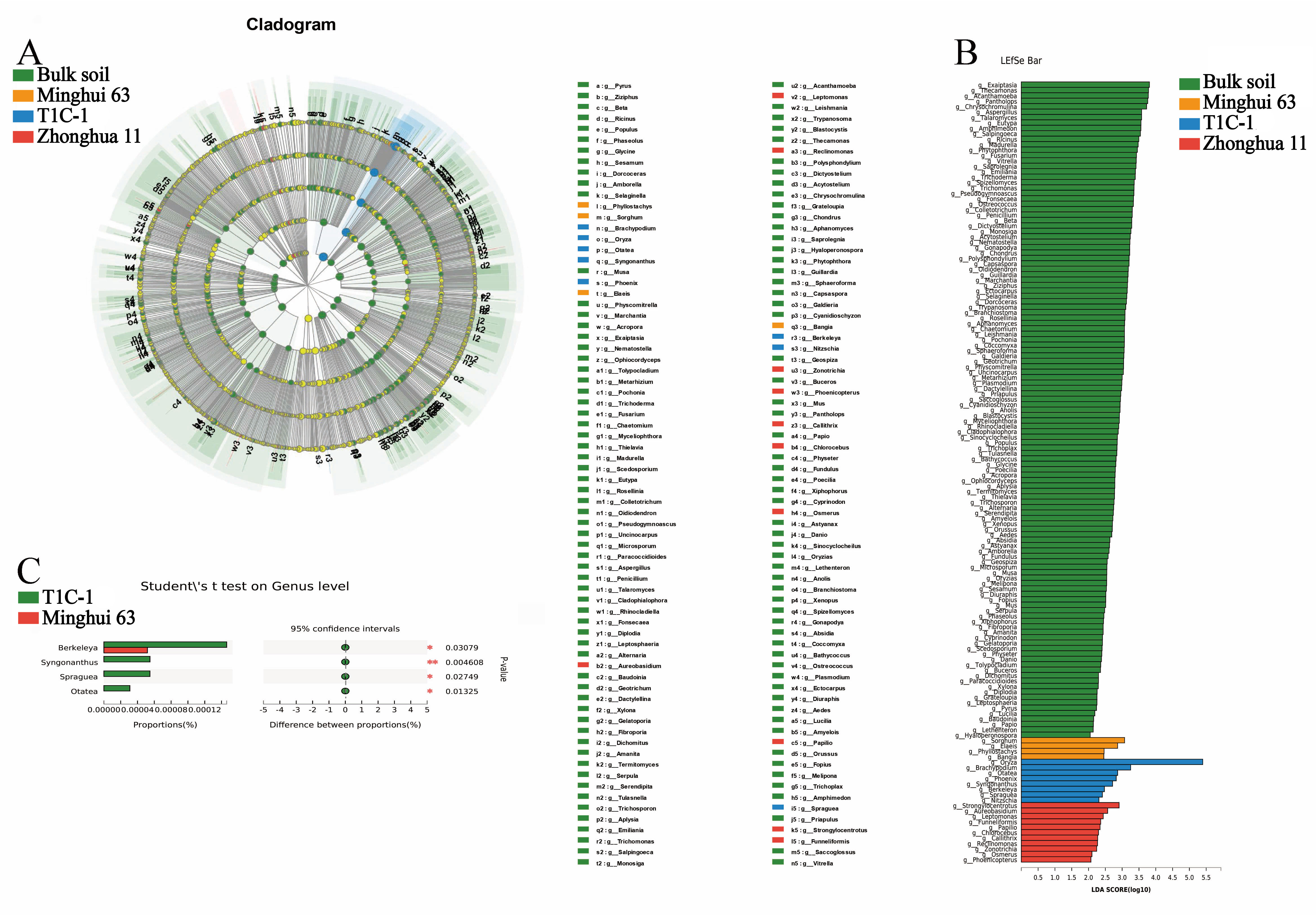


Supplementary Figure S11. LEfSe analysis of soil eukaryotic abundance. (A) Cladogram of soil eukaryotic communities. (B) LDA score identified the size of differentiation among three rice varieties and bulk soil control with a threshold value of 2.0. (C) Comparisons of eukaryotic abundances with significant differences between T1C-1 and Minghui 63.

**
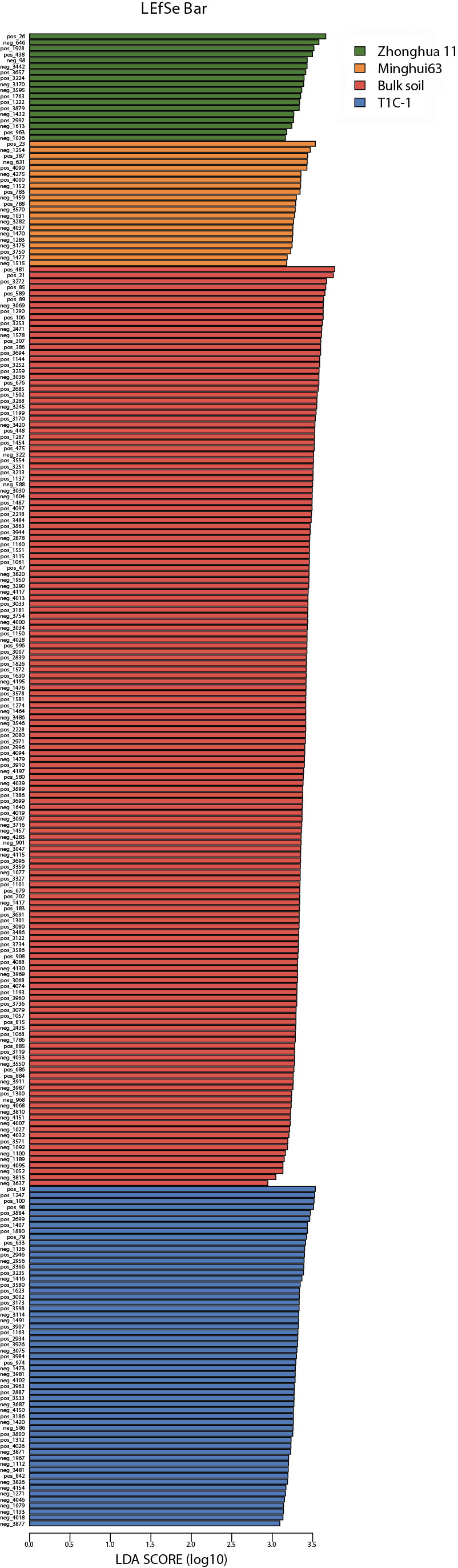
**

Supplementary Figure 12. LEfSe analysis of soil metabolites. LDA score identified the size of differentiation among three rice varieties and bulk soil control with a threshold value of 3.0.


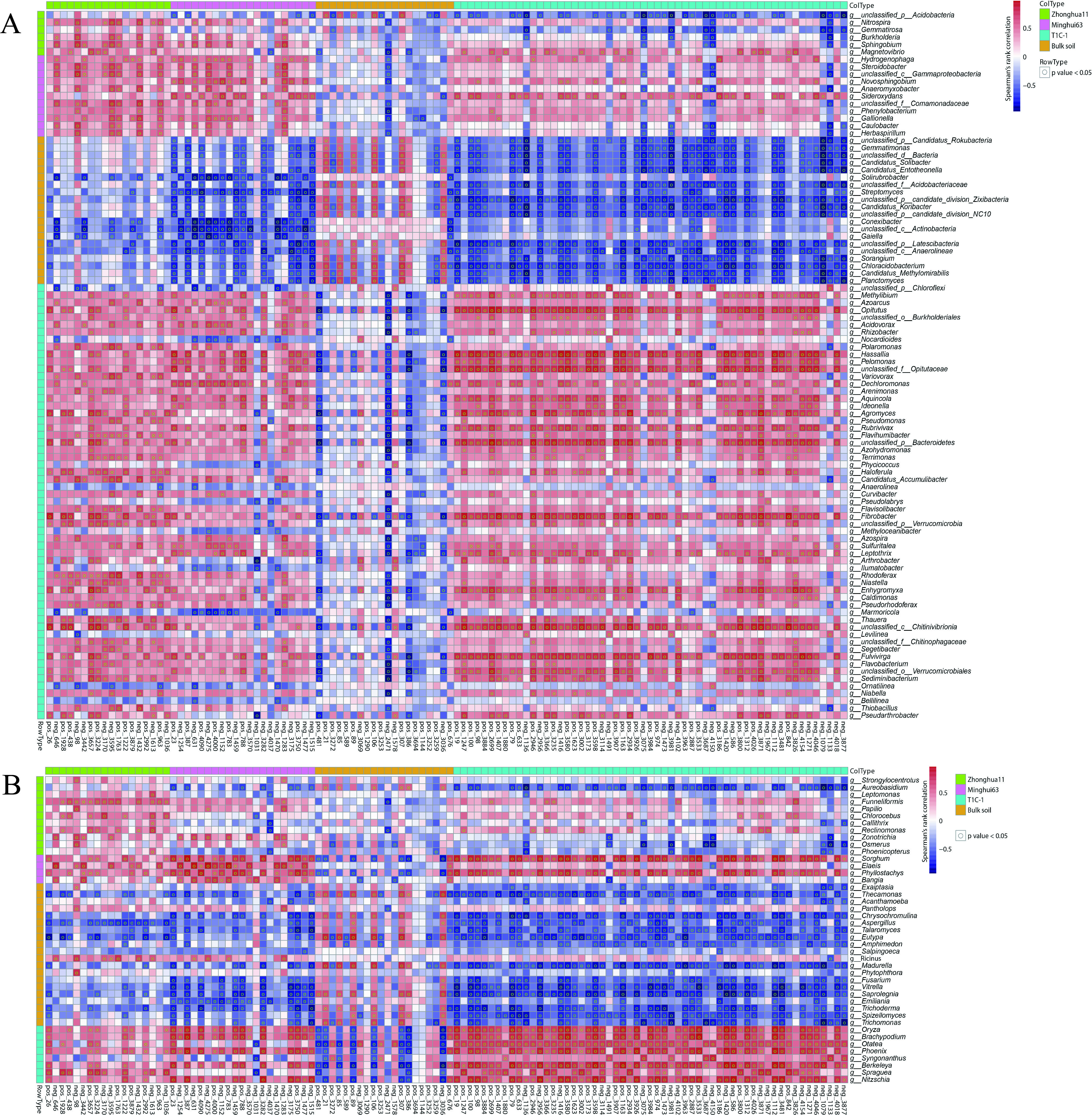


Supplementary Figure S13. Association between *Bt* rice-linked metabolites and bacteria (A) or eukaryota (B) .


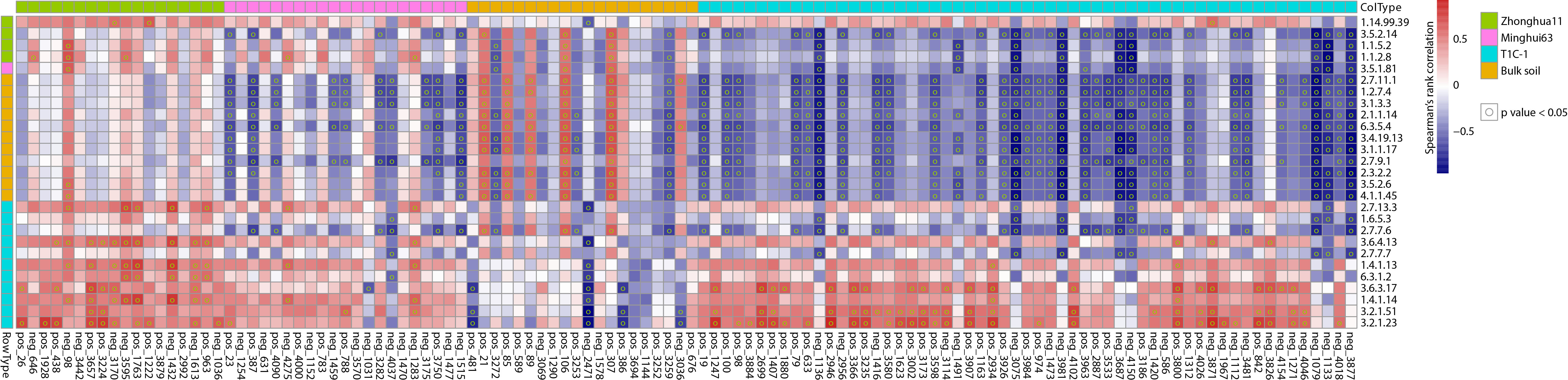


Supplementary Figure S14. Association between *Bt* rice-linked metabolites and microbial enzymes.


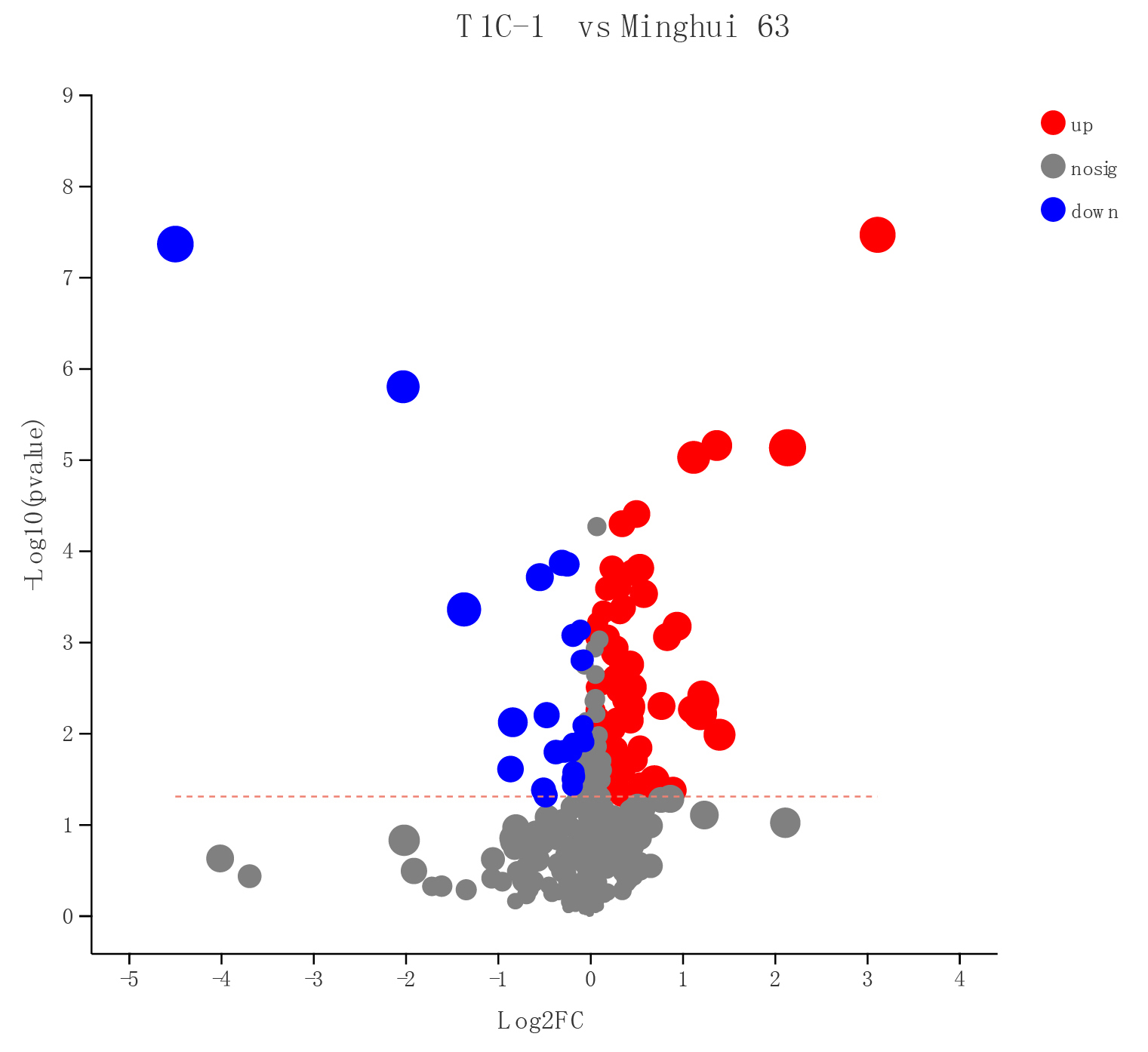


Supplementary Figure S15. Relative abundances of different soil metabolites annotated as standards. The volcano plot was obtained by plotting the log2 fold change on the x-axis and –log10 (*P*_value) on the y-axis. Metabolites that increased two fold or more with a *P* value <0.05 from a pair samples (T1C-1 vs Minghui 63) are indicated in red. Metabolites that decreased two fold or more with a *P* value <0.05 from a pair samples are indicated in blue. A total of metabolites colored by red and blue was 105. The other metabolites are indicated in gray.





Supplementary Figure S16. KEGG enrichment pathway of 105 soil representative metabolites belonging to T1C-1 compared with Minghui 63.

**

**

Supplementary Figure S17. Metabolomic analysis of rice root exudates. (A) Principal component analysis (PCA) of rice root exudate profiles based on metabolomic analyses and Bray-Curtis distances among samples. (B, C and D) Relative abundances of different metabolites. The volcano plot was obtained by plotting the log2 fold change on the x-axis and –log10 (*p* values) on the y-axis. Metabolites that differed two-fold or more between paired samples with a *p* < 0.05 are indicated in red. Metabolites that decreased two-fold or more with *p* < 0.05 from paired samples are indicated in green. The other metabolites are indicated in gray. (C) VIP-plot metabolite profiles of T1C-1 and Minghui 63 based on OPLS-DA analysis (VIP > 2, *p* < 0.05).


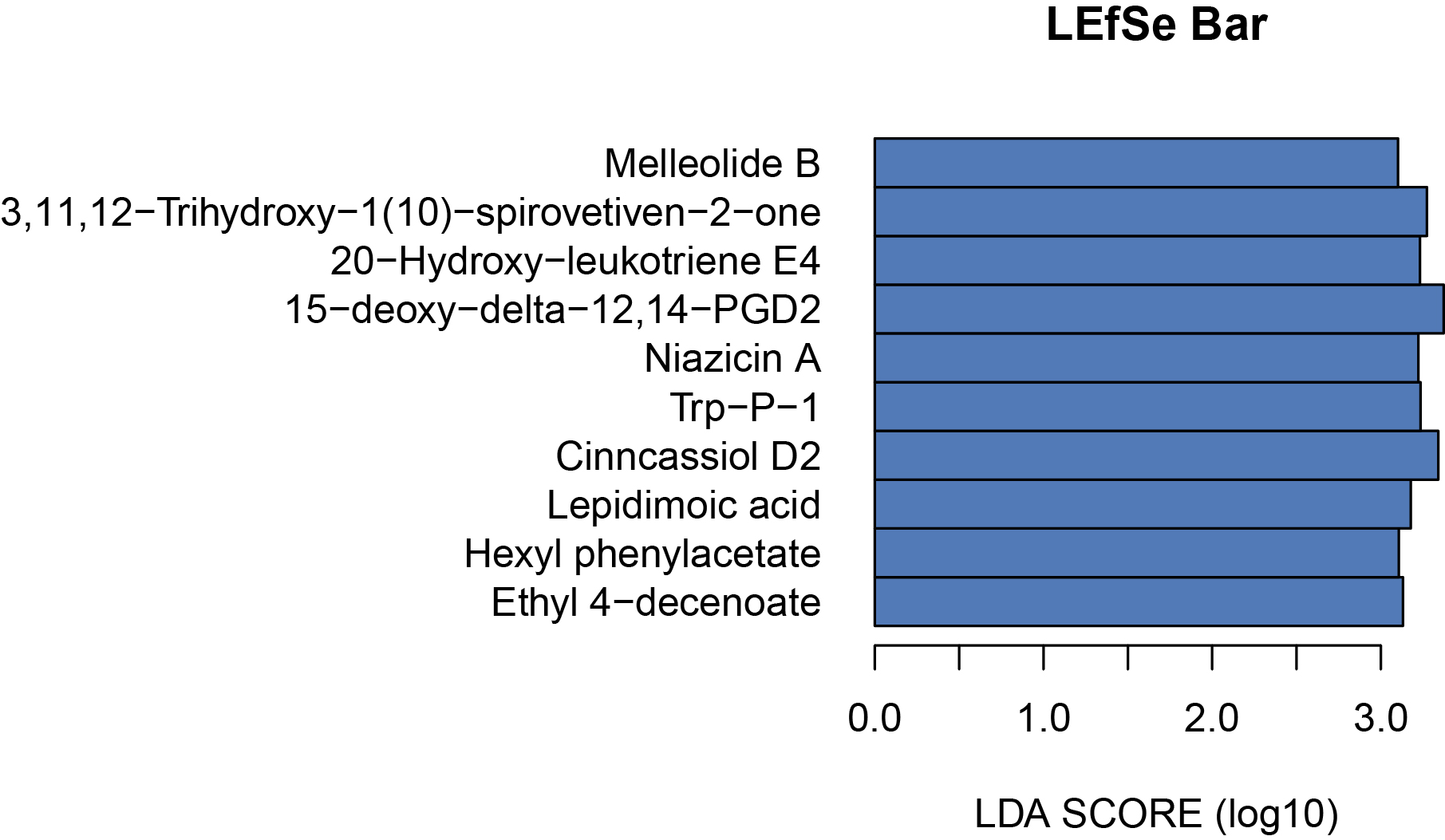


Supplementary Figure S18. LEfSe analysis of representative metabolites of T1C-1 in . root exudation metabolites. LDA score identified the size of differentiation with a threshold value of 3.0.

**
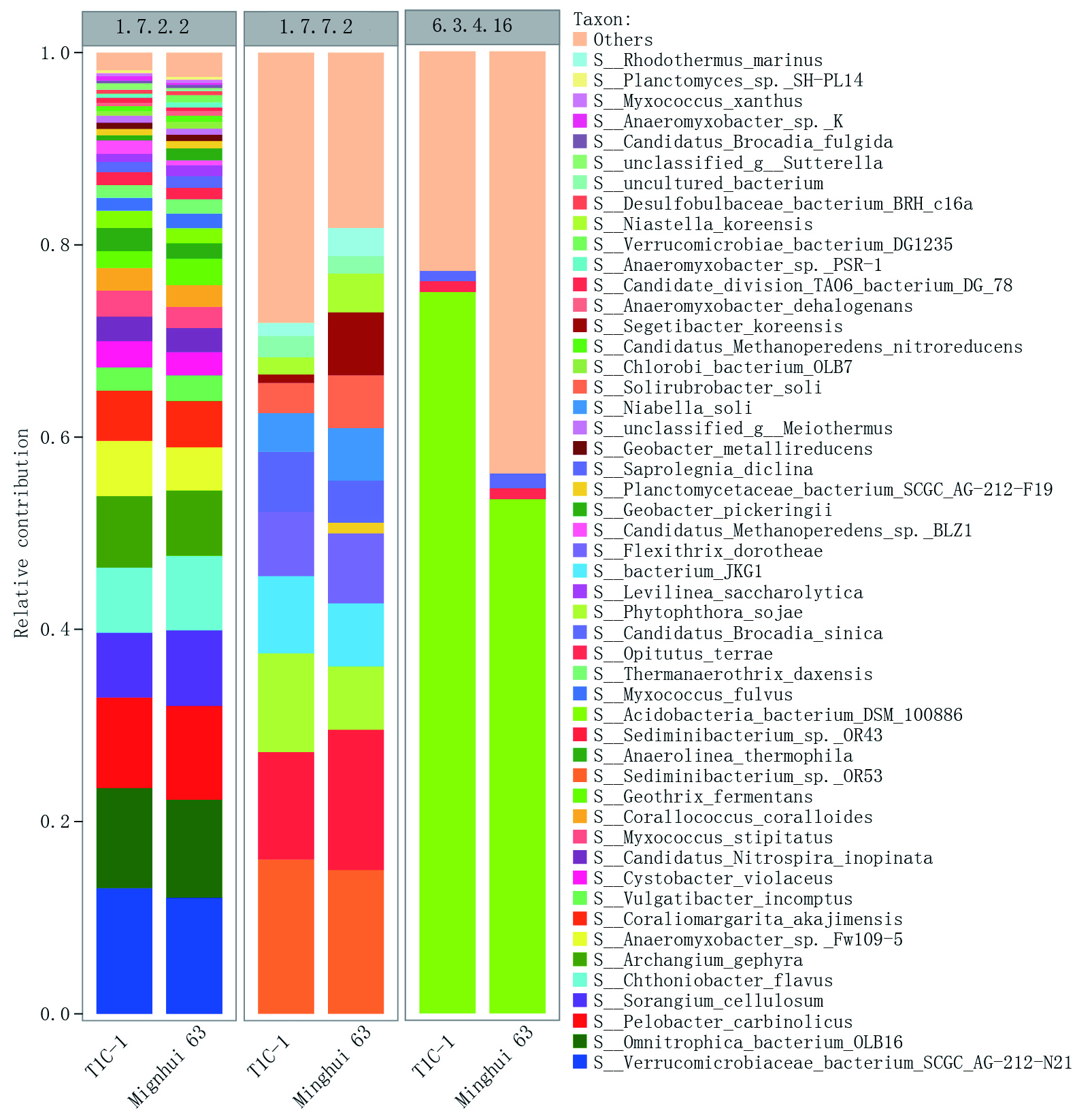
**

Supplementary Figure S19. Metagenomically contributed enzymes related to nitrogen metabolism that were differentially abundant in T1C-1 and Minghui 63, annotated by their species level contributors.


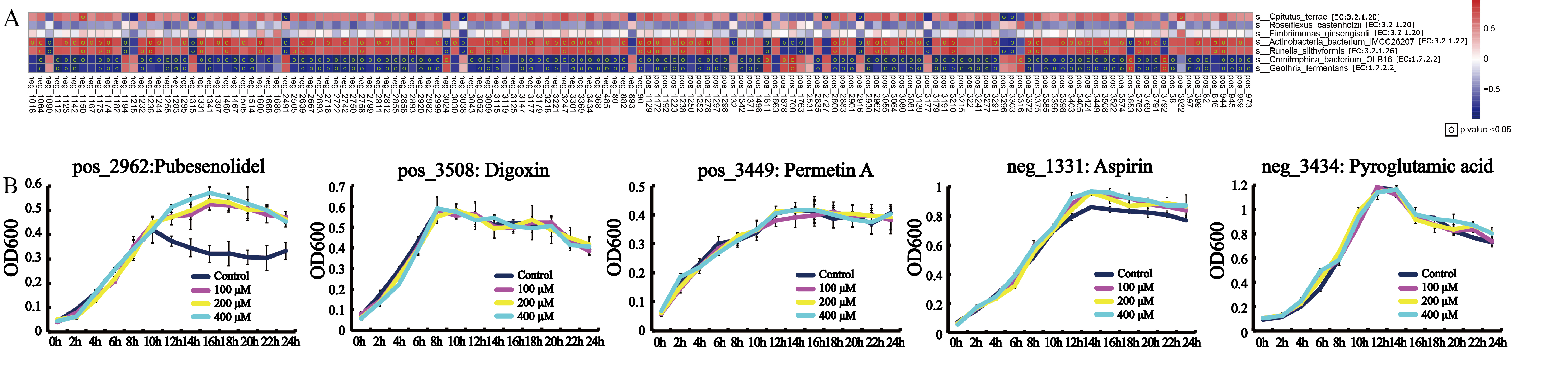


Supplementary Figure S20. Association between differentially abundant root exudation metabolites and soil microbial species related to C-N metabolism in T1C-1 and Minghui 63 (A), and validation of five predicted metabolite - *Runella slithyformis* relationships (B).


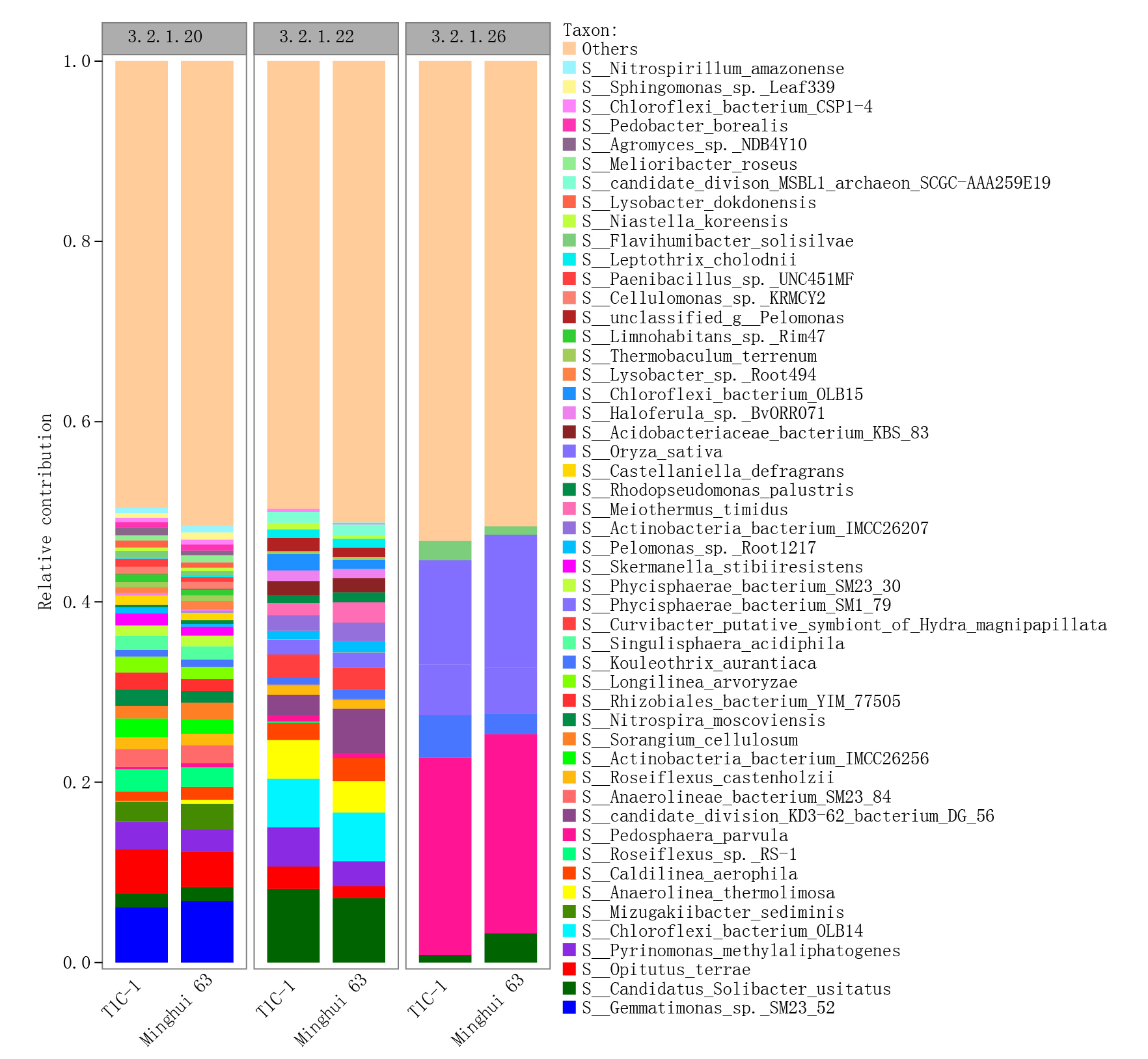


Supplementary Figure S21. Metagenomically contributed enzymes related to galactose metabolism that were differentially abundant in T1C-1 and Minghui 63, annotated by their species level contributors.


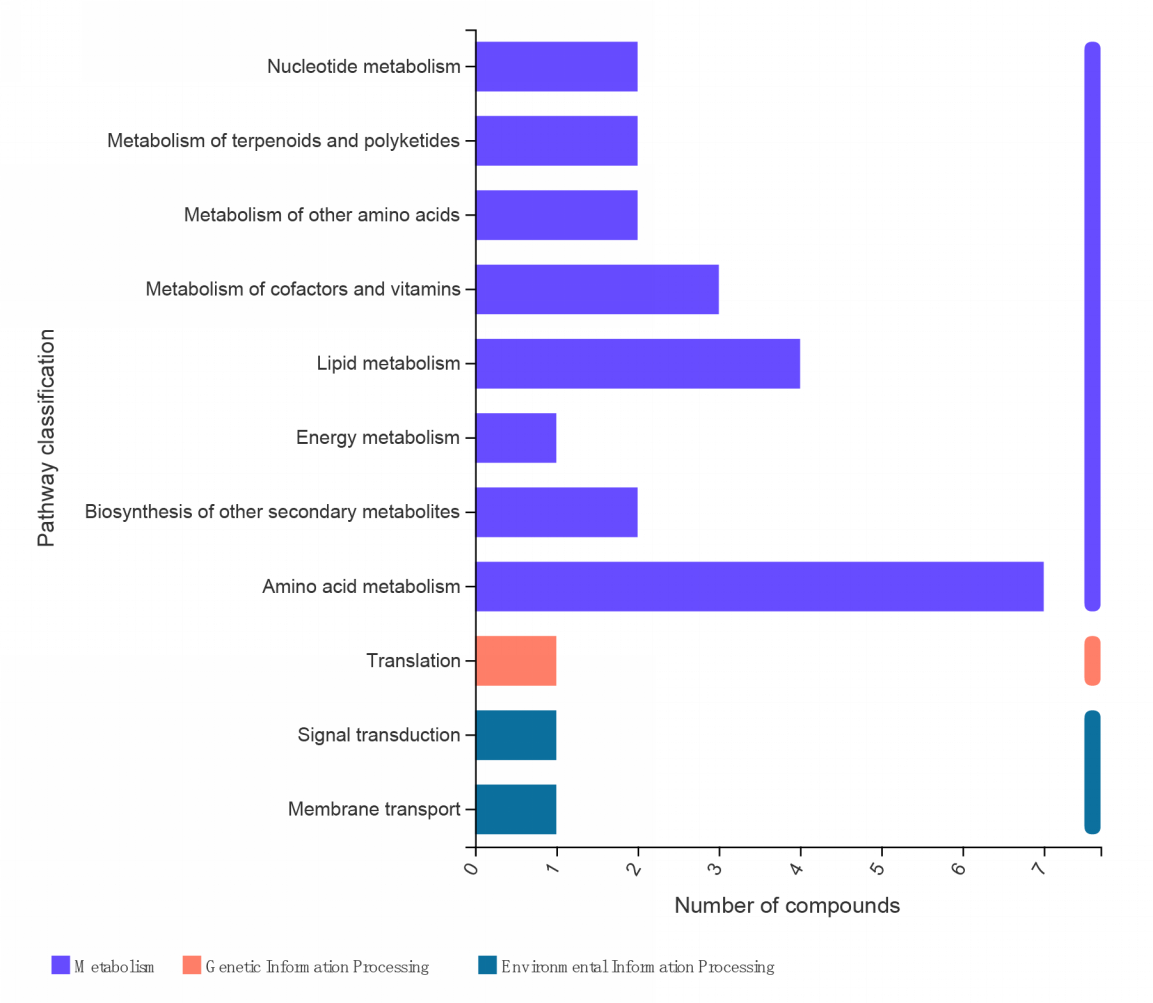


Supplementary Figure S22. KEGG enrichment pathway of differential root exudation metabolites between T1C-1 and Minghui 63.
